# Supplementary material for: Data-centric artificial olfactory system based on the eigengraph
Source: Nat Commun. 2024 Feb 8;15:1211. doi: 10.1038/s41467-024-45430-9 (PMC10853498; doi:10.1038/s41467-024-45430-9)
Supplement: Supplementary file 1 — Supplementary Information [file 41467_2024_45430_MOESM1_ESM.pdf]

Supplementary information

Data-centric artificial olfactory system based on the eigengraph

Seung-Hyun Sung[1,2,8], Jun Min Suh[3,4,8], Yun Ji Hwang[1], Ho Won Jang[3,5,9], Jeon Gue Park[6,7,9] and Seong Chan Jun[1,9]

[1]School of Mechanical Engineering, Yonsei University, Seoul 03722, Republic of Korea.

[2]Finance Division, Daejeon Metropolitan Office of Education, Daejeon 35239, Republic of Korea.

[3]Department of Materials Science and Engineering, Research Institute of Advanced Materials, Seoul National University, Seoul 08826, Republic of Korea.

[4]Department of Mechanical Engineering, Massachusetts Institute of Technology, Cambridge, MA 02139, United States.

[5]Advanced Institute of Convergence Technology, Seoul National University, Suwon 16229, Republic of Korea

[6]Artificial Intelligence Laboratory, Tutorus Labs Inc., Seoul 06595, Republic of Korea.

[7]Center for Educational Research, College of Education, Seoul National University, Seoul 08826, Republic of Korea.

[8]These authors contributed equally: Seung-Hyun Sung, Jun Min Suh.

[9]These authors jointly supervised this work: Ho Won Jang, Jeon Gue Park, Seong Chan Jun. e-mail: hwjang@snu.ac.kr; jgpark@tutoruslabs.com; scj@yonsei.ac.kr

Table of contents

Supplementary Fig. 1. Cross-sectional and top-view (inset) scanning electron microscopy (SEM) images of all sensing channels of the olfactory receptor-like sensor array

Supplementary Fig. 2. X-ray diffraction (XRD) patterns of the all channels of the olfactory receptor-like sensor array

Supplementary Fig. 3. X-ray photoelectron spectroscopy (XPS) and transmission electron spectroscopy (TEM) images of NiO-based channels

Supplementary Fig. 4. The database based on the three indistinguishable gas molecules for deep learning analysis

Supplementary Fig. 5. Frequency-domain power spectra with linear frequency bins

Supplementary Fig. 6. 4-inch standard wafer with integrated electrode patterns for mass production of olfactory receptor-like sensor arrays through semiconductor processing

Supplementary Fig. 7. Gas measurement and monitoring system

Supplementary Fig. 8. A circuit schematic of a multi-measurement system for a 3x3 sensor array

Supplementary Fig. 9. Certified reference materials for the automobile exhaust gases that have been manufactured and verified through metrologically valid and strict procedures by KOLAS (Korean Laboratory Accreditation Scheme)

Supplementary Fig. 10. A total of four sets of time series eigengraphs for exhaust gases generated from the different four ORSA chips simultaneously fabricated in the same manufacturing process

Supplementary Fig. 11. Training result curves of the 4-fold cross validation for the empirical experiments of the exhaust gases using the three deep learning architecture (CNN, DNN and CNN-LSTM)

Supplementary Table 1. Technical specifications of electron beam evaporation to fabricate the olfactory receptor-like sensor array

Supplementary Table 2. Theoretical detection limits of all channels of the olfactory receptor-like sensor array for the indistinguishable three gas molecules through principal component analysis

Supplementary Table 3. Class definition and ID assignment of the 117 datasets for all channels of the olfactory receptor-like sensor array according to the gas species and mixing ratios using binary representation

Supplementary Table 4. Summary of deep neural network (DNN) architecture which has fully

connected input layer, output layer and 3 hidden layers

Supplementary Table 5. Summary of variables setting and results for eigengraph deep learning analysis

Supplementary Table 6. Euclidean distance for similarity analysis between all measurement data of intra-class for the ATX singular and mixed gases

Supplementary Table 7. Euclidean distance for similarity analysis between all measurement data of intra-class for the exhaust gases and their individual components

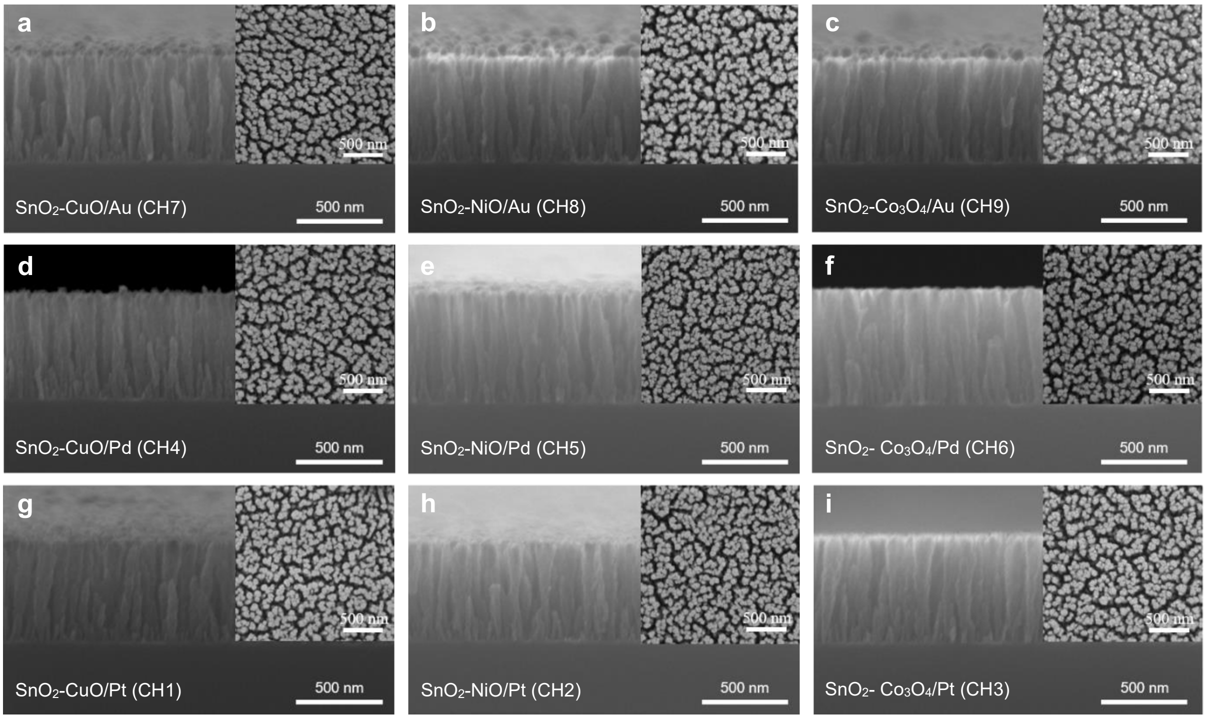

Supplementary Fig. 1. Cross-sectional and top-view (inset) scanning electron microscopy (SEM) images of all sensing channels of the olfactory receptor-like sensor array. a, SnO_2-CuO/Au. **b,** SnO_2-NiO/Au. **c,** SnO_2-Co_3O_4/Au. **d,** SnO_2-CuO/Pd. **e,** SnO_2-NiO/Pd. **f,** SnO_2- Co_3O_4/Pd. **g,** SnO_2-CuO/Pt. **h,** SnO_2-NiO/Pt. and **i,** SnO_2- Co_3O_4/Pt.

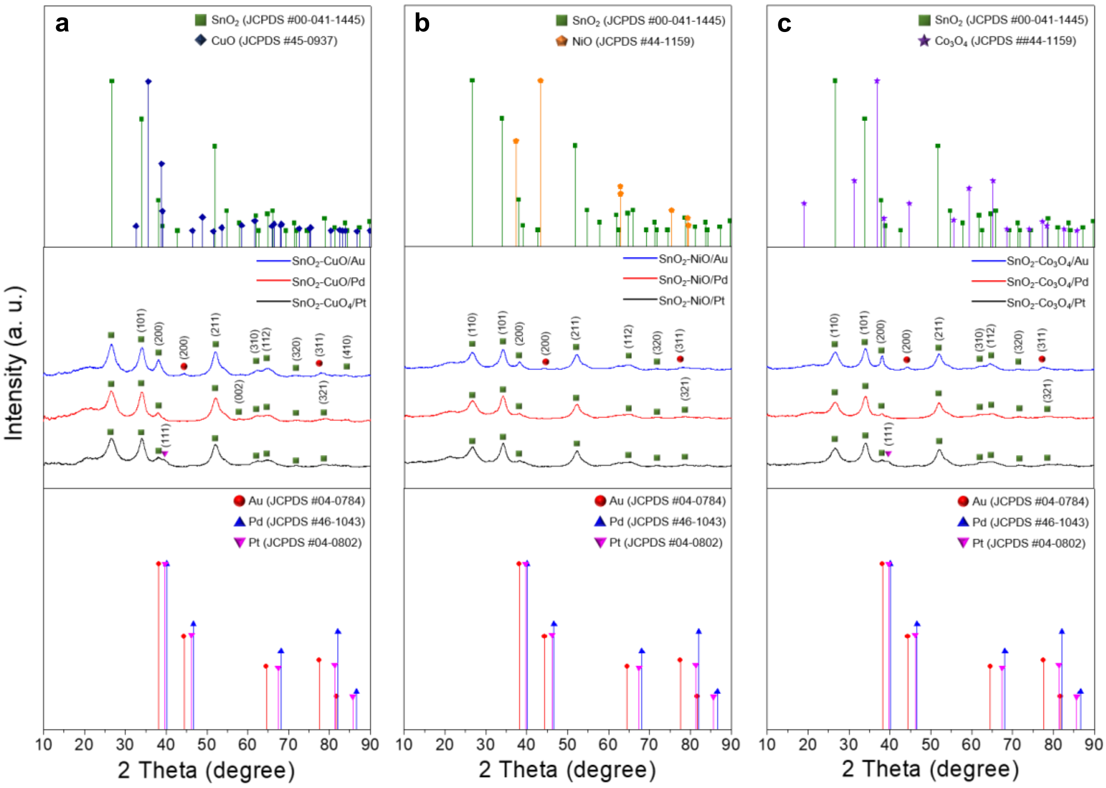

Supplementary Fig. 2. X-ray diffraction (XRD) patterns of the all channels of the olfactory receptor-like sensor array. a, CuO-based 3 channels (CH1, CH4, CH7), **b,** NiO-based 3 channels (CH2, CH5, CH7), and **c,** Co_3O_4-based 3 channels (CH3, CH6, CH9).

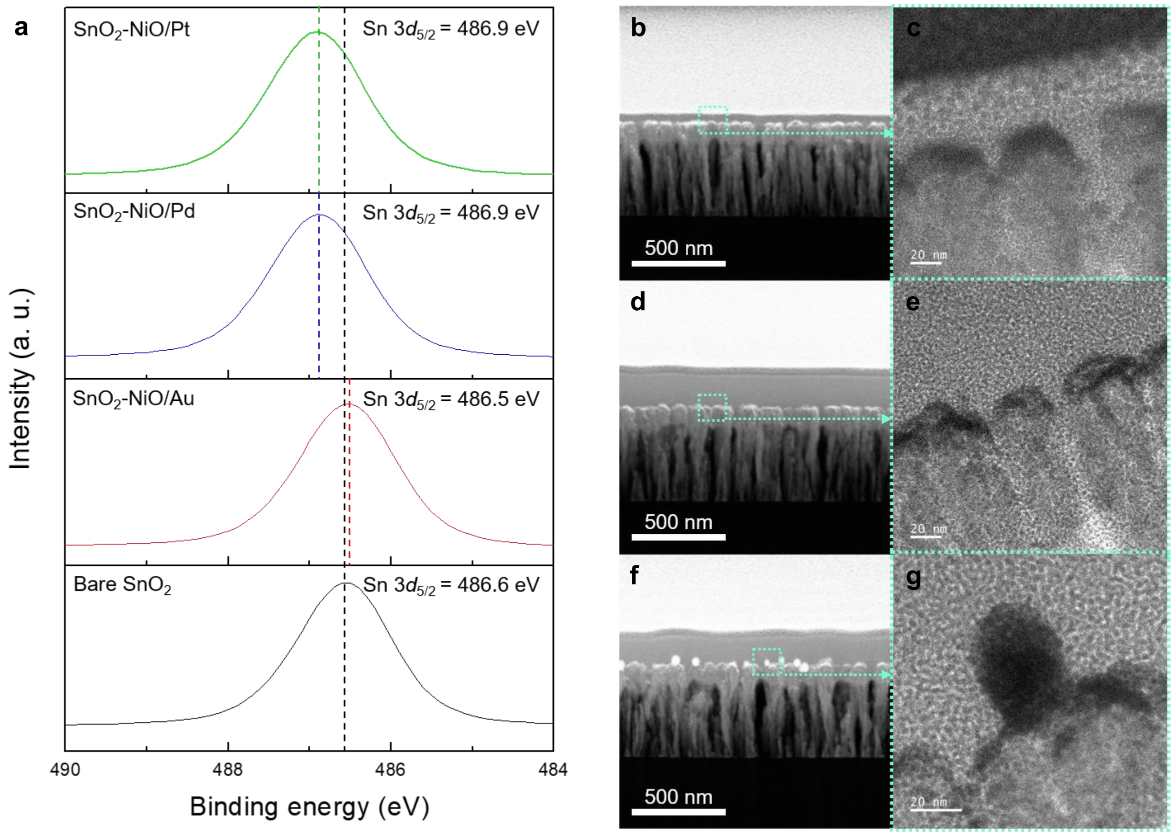

Supplementary Fig. 3. X-ray photoelectron spectroscopy (XPS) and transmission electron spectroscopy (TEM) images of NiO-based channels. a, Investigation of electron transfer between the composite nanocatalysts and SnO_2 nanorods based on the binding energy shift. Cross-sectional TEM images of **b,** SnO_2-NiO/Pt. **d,** SnO_2-NiO/Pd. **f,** SnO_2-NiO/Au. Morphological TEM images of the composite nanocatalyst **c,** NiO/Pt. **e,** NiO/Pd. **g,** NiO/Au.

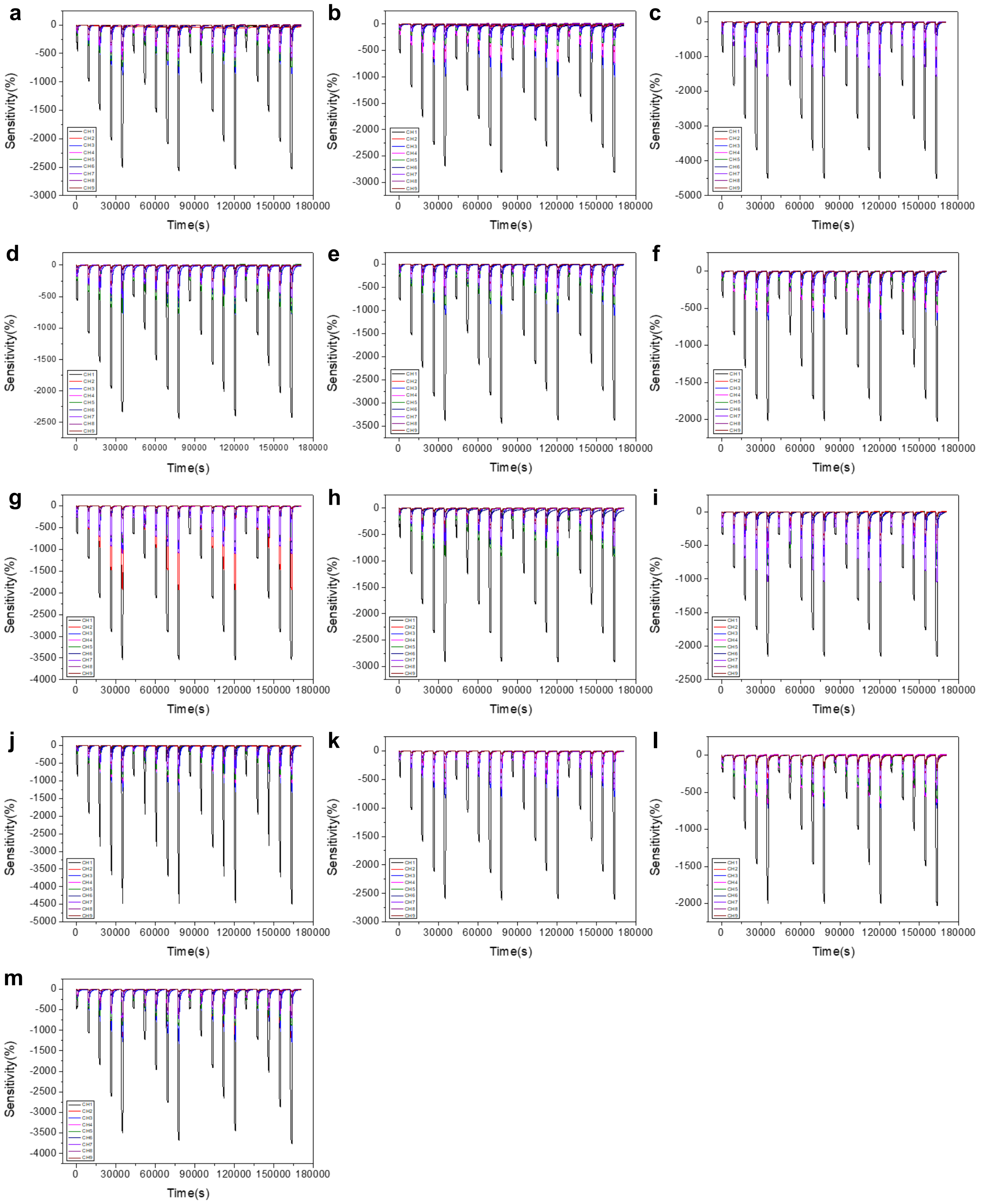

Supplementary Fig. 4. The database based on the three indistinguishable gas molecules for deep learning analysis. Long-term response curves to **a,** Acetone, **b,** Toluene, **c,** Xylene, **d,** AT11, **e,** AT13, **f,** AT31, **g,** AX11, **h,** AX13, **i,** AX31, **j,** TX11, **k,** TX13, **l,** TX31 and **m,** ATX111. The sum of concentrations of each gas molecule constituting the mixed gases was 2, 4, 6, 8, and 10 ppm. (A: acetone, T: toluene, X: xylene, the latter numbers of 11, 13, 31 and 111 mean the mixing ratios of the constituent gas molecules).

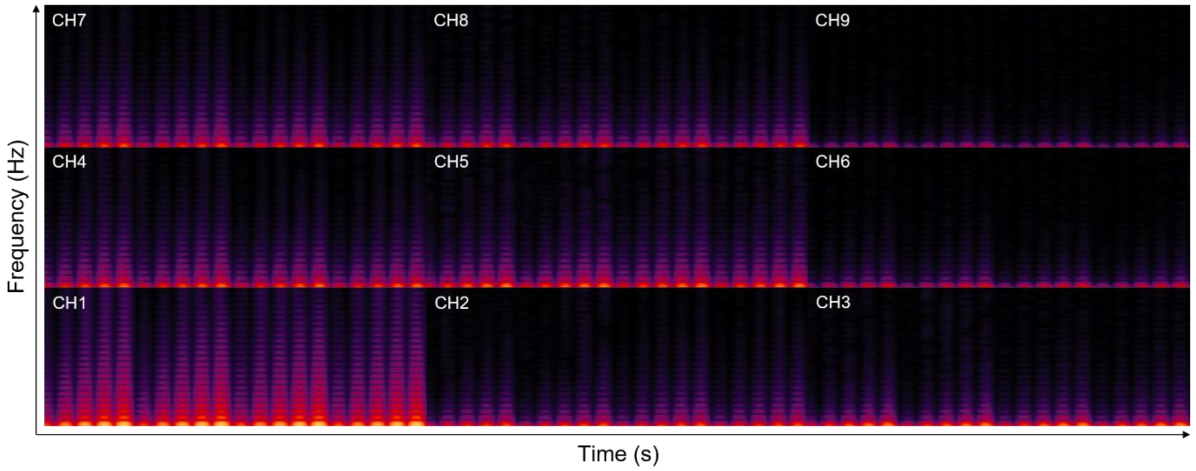

Supplementary Fig. 5. Frequency-domain power spectra with linear frequency bins. Time-series response signals to the acetone gas of all channels of the olfactory receptor-like sensor array were converted into the power spectra based on the fast Fourier transform algorithm.

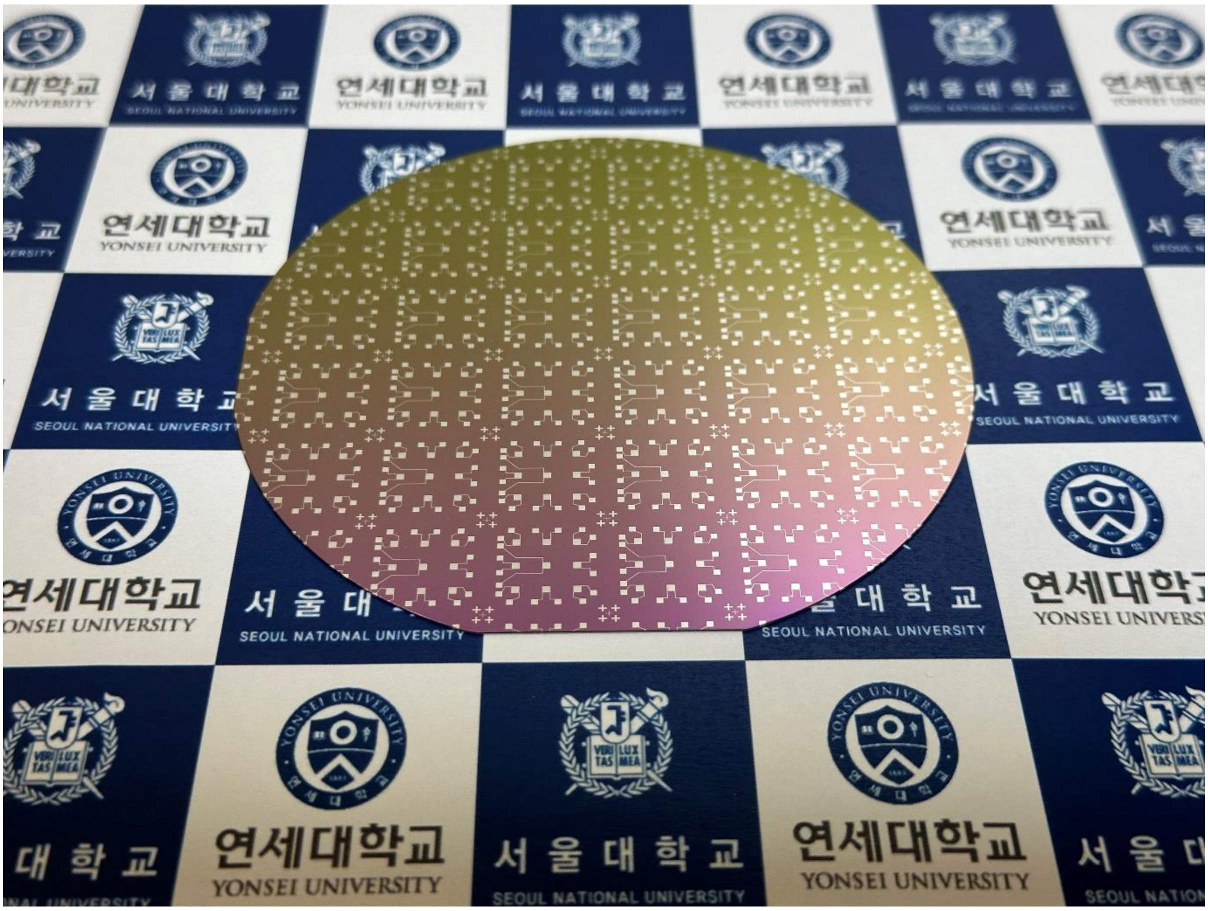

Supplementary Fig. 6. 4-inch standard wafer with integrated electrode patterns for mass production of olfactory receptor-like sensor arrays through semiconductor processing. The Si/SiO_2 wafer (300 nm / 525 μm thickness) was processed with 99.999999999% pure HF (DS Semicon Co., Ltd.), and the high-purity process is effective in preventing leakage current and noises due to the presence of impurities in advances.

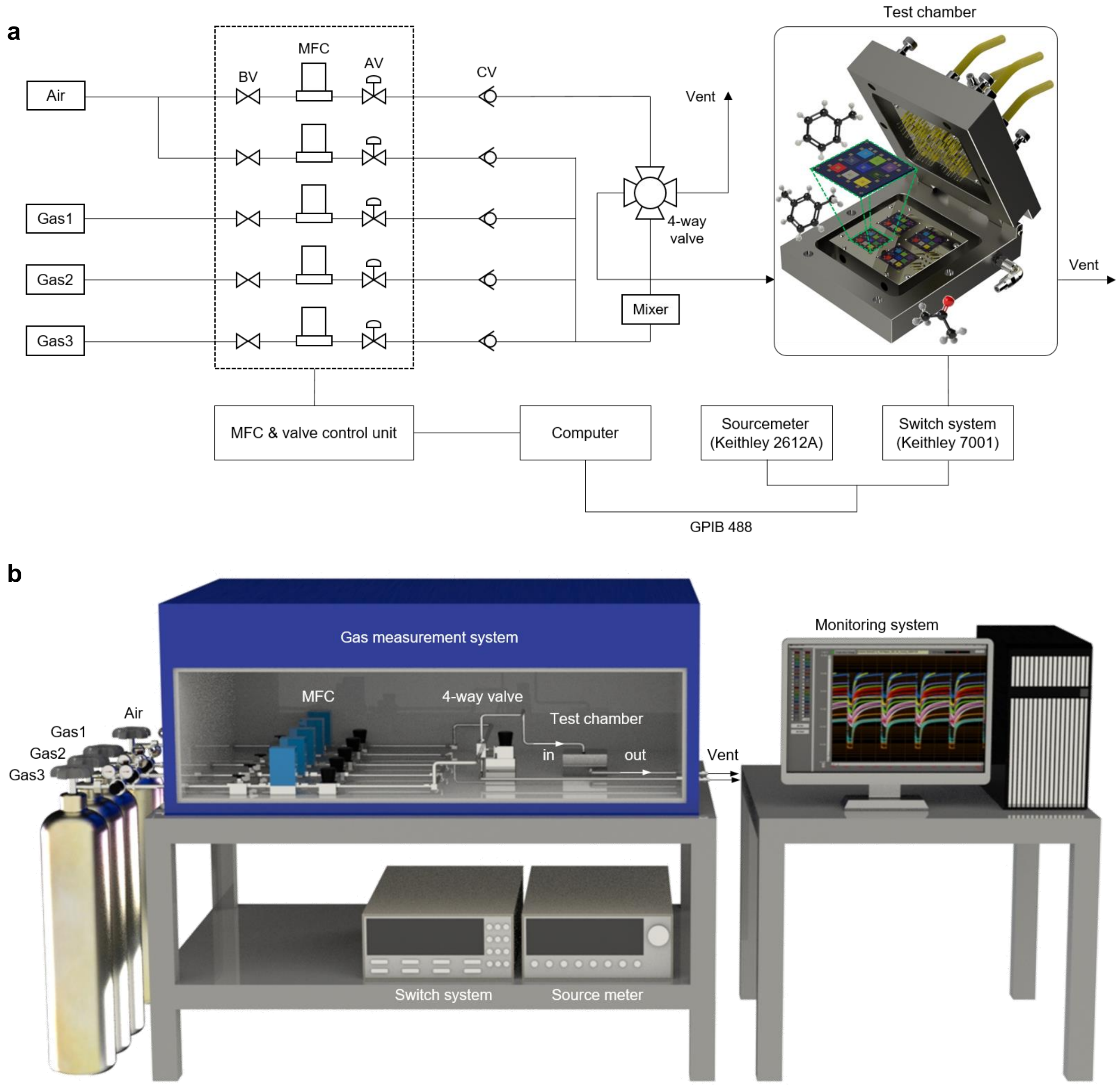

Supplementary Fig. 7. Gas measurement and monitoring system. a, Schematic diagram and **b,** 3D configuration of the system. The gas supply unit has 1 air and 3 target gas flow paths, and the target concentration can be adjusted by mixing air with the target gas. The measurement and monitoring system can accommodate up to four sensor array chips and simultaneously monitor the gas response of the total 36 channels.

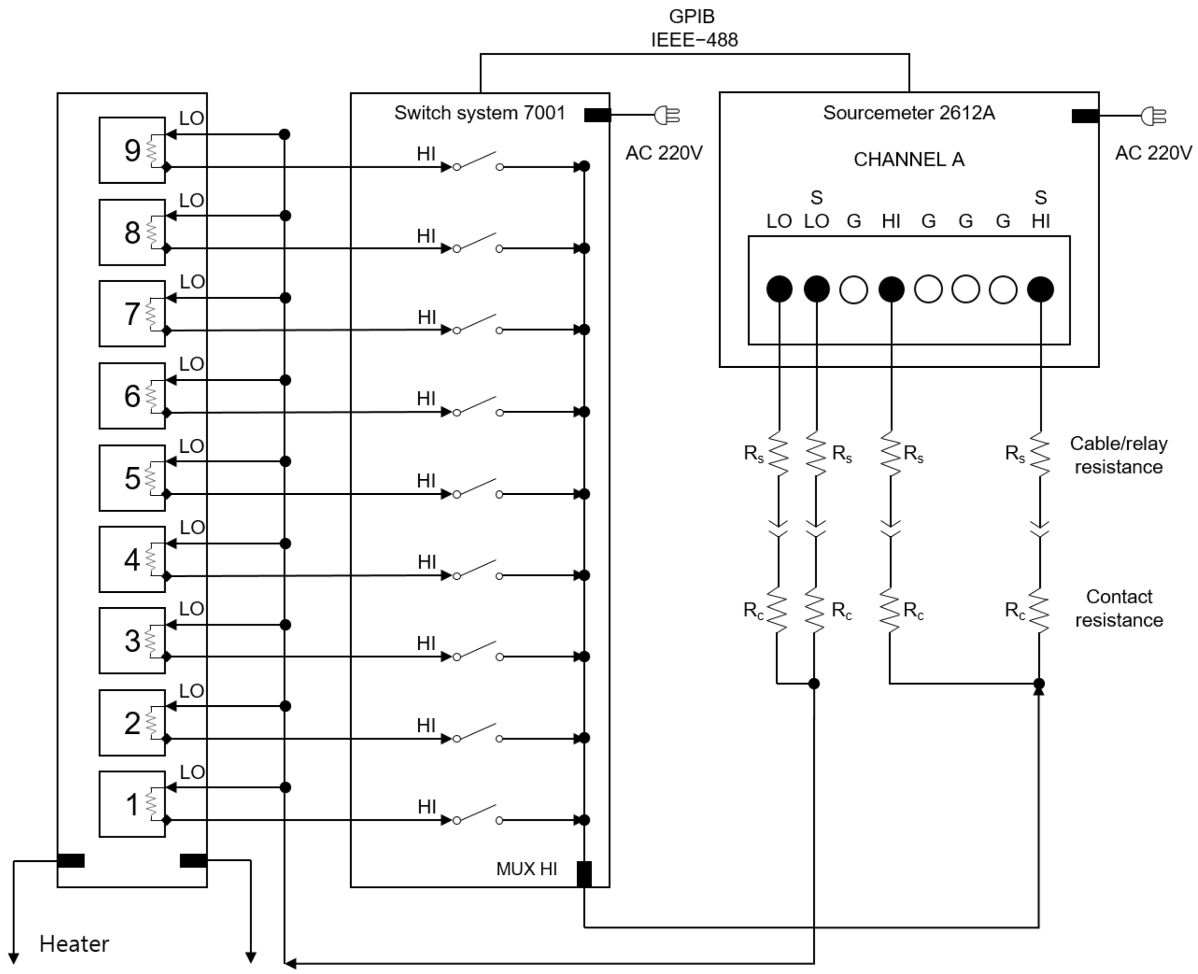

Supplementary Fig. 8. A circuit schematic of a multi-measurement system for a 3x3 sensor array. The switch system sequentially relays the switches assigned to each sensing channel to transmit the electrical signals to the sourcemeter which measures the ohmic values of the signals.

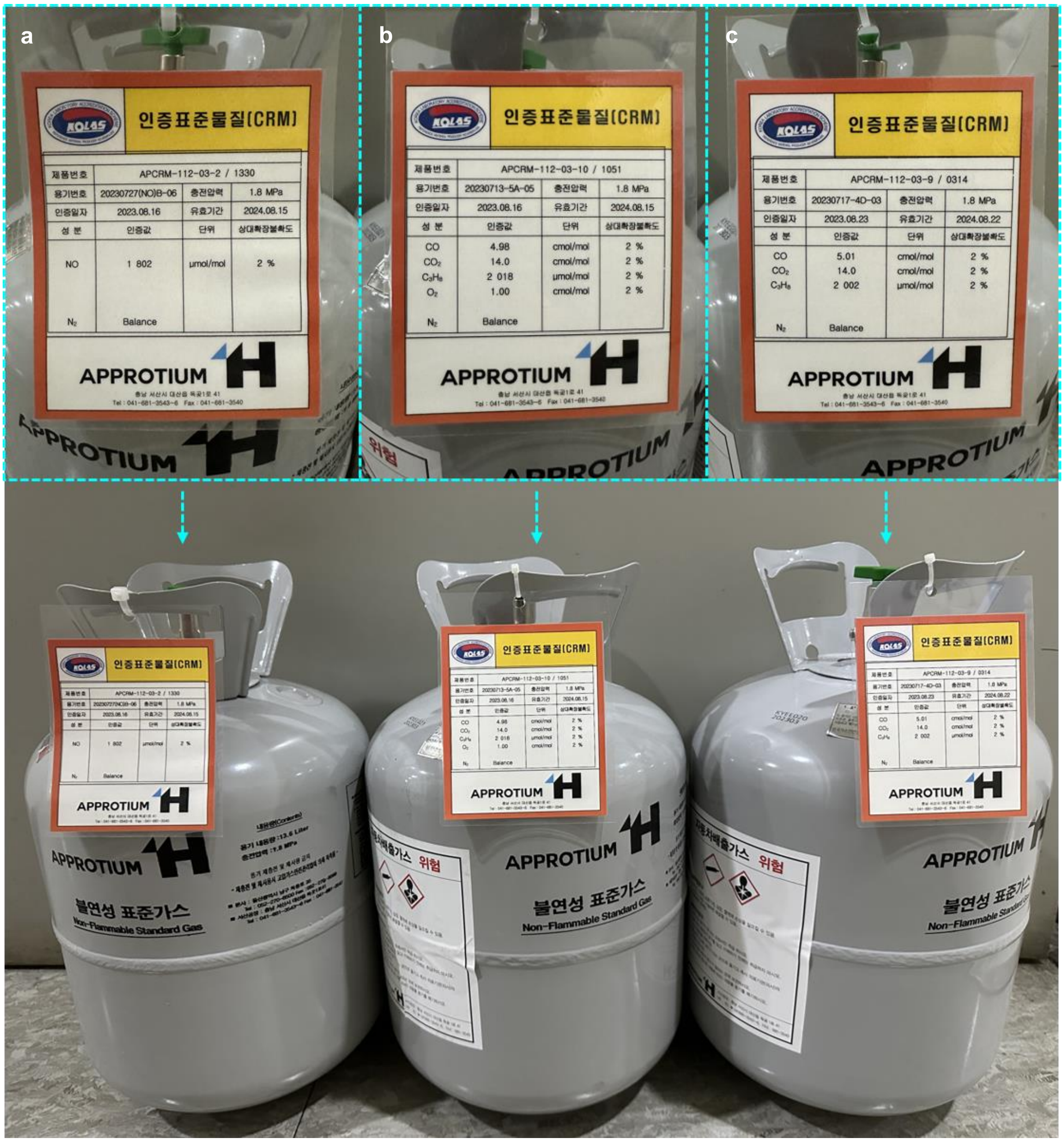

Supplementary Fig. 9 | Certified reference materials for the automobile exhaust gases that have been manufactured and verified through metrologically valid and strict procedures by KOLAS (Korean Laboratory Accreditation Scheme). a, b Standard exhaust gases for legally mandated emissions testing for diesel engines including NO (0.18%), CO (4.98%), CO_2 (14.0%), C_3H_8 (0.20%) and O_2 (1 %) **c**, Standard exhaust gases for legally mandated emissions testing for gasoline engines including CO (5.01%), CO_2 (14.0%) and C_3H_8 (0.20%). The accompanying standard material certificates guarantee the content and relative expanded uncertainty of each component for precise measurements.

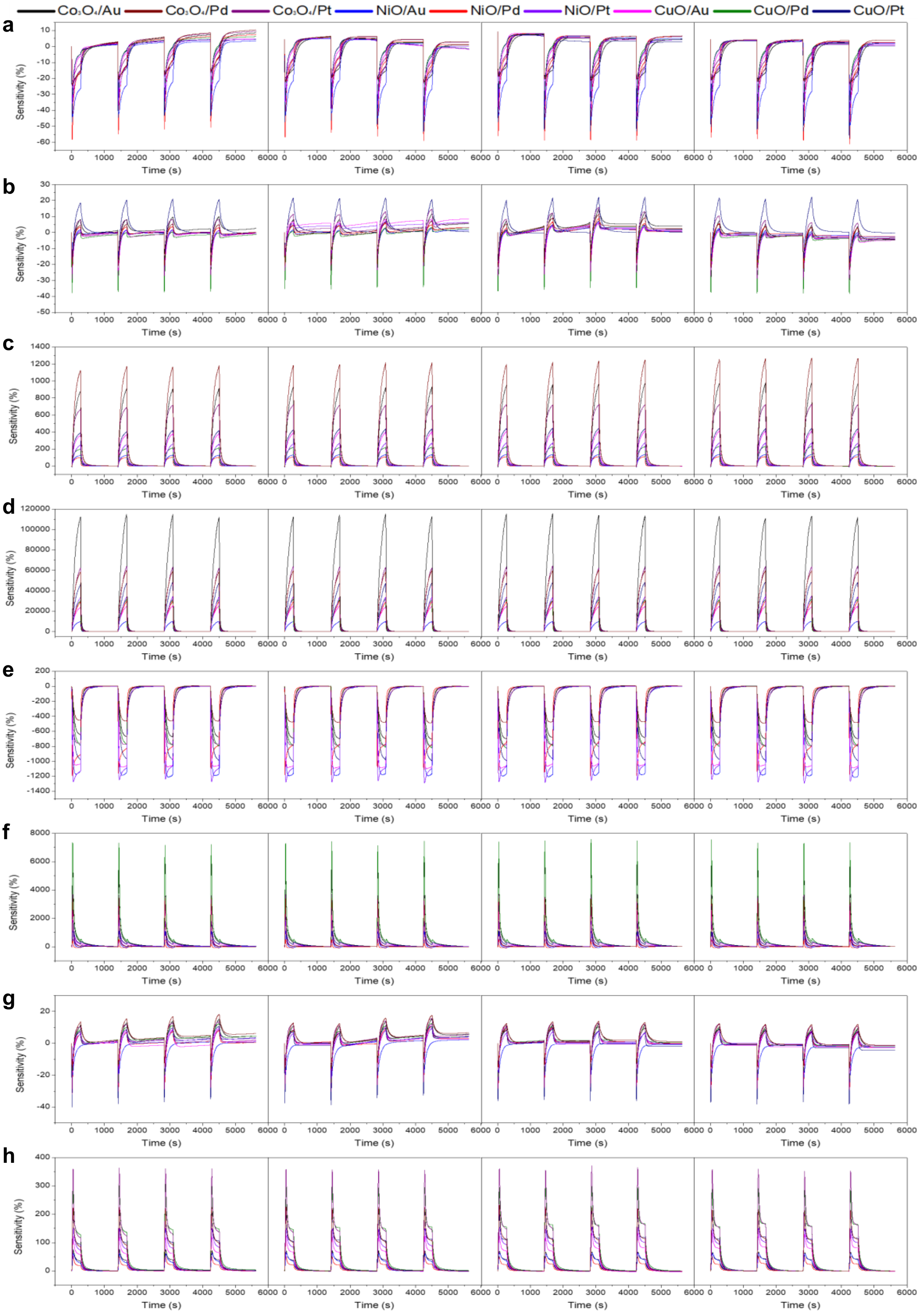

Supplementary Fig. 10 | A total of four sets of time series eigengraphs for exhaust gases generated from the different four ORSA chips simultaneously fabricated in the same manufacturing process. a, CO. **b,** CO_2. **C,** NO. **d,** NO_2. **e,** Gasoline exhaust gas (including CO(5.01 %), CO_2(14.0 %), C_3CH_8(0.20 %)). **f,** Diesel exhaust gas(including NO(0.18 %), CO(4.98 %), CO_2(14.0 %), C_3CH_8(0.20 %) and O_2(1 %)). **g,** CO+CO_2. **h,** CO+CO_2+NO.

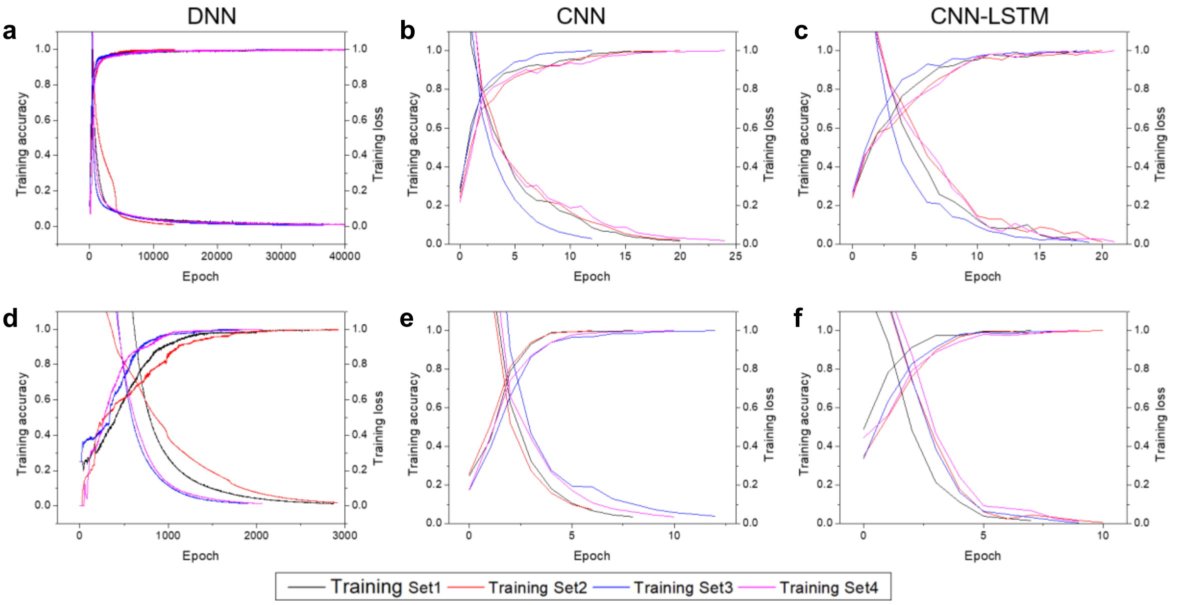

Supplementary Fig. 11 | Training result curves of the 4-fold cross validation for the empirical experiments of the exhaust gases using the three deep learning architecture (CNN, DNN and CNN-LSTM). Training accuracy and training loss graphs using **a**, DNN, **b**, CNN and **c**, CNN-LSTM for the two exhaust gases and four individual components. Training accuracy and training loss graphs using **d**, DNN, **e**, CNN and **f**, CNN-LSTM for four mixed gases including $CO+CO_2$, $CO+CO_2+NO$, gasoline and diesel exhaust gases.

Supplementary Table 1. Technical specifications of electron beam evaporation to fabricate the olfactory receptor-like sensor array.

Target	Glancing angle	Rotation speed	Base pressure	Deposition pressure	Growth rate	Thickness
SnO_2	80º	80 rpm	4.4×10^{-7}	4.3×10^{-5}	1.5 ~ 2.0 Å/s	500 nm
Cu	0º	No rotation	3.0×10^{-6}	Pass torr	0.04 Å/s	1.0 nm
Ni	0º	No rotation	1.1×10^{-6}	3.7×10^{-6}	0.03 Å/s	1.0 nm
Co	0º	No rotation	1.0×10^{-6}	3.3×10^{-6}	0.03 ~ 0.04 Å/s	1.0 nm
Au	0º	No rotation	1.6×10^{-6}	4.8×10^{-6}	0.04 Å/s	1.0 nm
Pd	0º	No rotation	2.0×10^{-6}	3.7×10^{-6}	0.06 Å/s	1.0 nm
Pt	0º	No rotation	1.7×10^{-6}	6.0×10^{-6}	0.03 Å/s	1.0 nm

Supplementary Table 2. Theoretical detection limits of all channels of the olfactory receptor-like sensor array for the indistinguishable three gas molecules through principal component analysis.

Channel		CH1	CH2	CH3	CH4	CH5	CH6	CH7	CH8	CH9
Acetone	rms	0.6899	0.05936	0.1194	0.2809	0.07723	0.01967	0.06851	0.03011	0.007618
	slope	249.4280	54.08405	104.08854	61.2680	38.3320	22.1546	24.9290	9.9935	5.3224
	DL (ppb)	8.2982	3.2928	3.4433	13.7556	6.04483	2.6642	8.2470	9.03792	4.2904
Toluene	rms	0.3833	0.01597	0.007352	0.07738	0.02759	0.01307	0.04113	0.01453	0.01341
	slope	249.9857	46.3132	92.05123	61.7451	37.2128	21.1644	47.01643	22.9712	8.5229
	DL (ppb)	4.6001	1.03489	0.2396	3.7597	2.2249	1.8531	2.6246	1.8981	4.7214
Xylene	rms	0.3468	0.03765	0.1165	0.1677	0.1031	0.03248	0.06930	0.01793	0.01491
	slope	456.5366	77.4479	139.4088	95.8966	57.57	30.6187	86.3346	36.5372	8.0903
	DL (ppb)	2.2794	1.4585	2.5084	5.2482	5.3771	3.1832	2.4081	1.4722	5.5321

Supplementary Table 3. Class definition and ID assignment of the 117 datasets for all channels of the olfactory receptor-like sensor array according to the gas species and mixing ratios using binary representation.

Class	CH1	CH2	CH3	CH4	CH5	CH6	CH7	CH8	CH9
A	1<br>(1)	2<br>(10)	3<br>(11)	4<br>(100)	5<br>(101)	6<br>(110)	7<br>(111)	8<br>(1000)	9<br>(1001)
T	11<br>(1011)	12<br>(1100)	13<br>(1101)	14<br>(1110)	15<br>(1111)	16<br>(10000)	17<br>(10001)	18<br>(10010)	19<br>(10011)
X	21<br>(10101)	22<br>(10110)	23<br>(10111)	24<br>(11000)	25<br>(11001)	26<br>(11010)	27<br>(11011)	28<br>(11100)	29<br>(11101)
AT11	31<br>(11111)	32<br>(100000)	33<br>(100001)	34<br>(100010)	35<br>(100011)	36<br>(100100)	37<br>(100101)	38<br>(100110)	39<br>(100111)
AT13	41<br>(101001)	42<br>(101010)	43<br>(101011)	44<br>(101100)	45<br>(101101)	46<br>(101110)	47<br>(101111)	48<br>(110000)	49<br>(110001)
AT31	51<br>(110011)	52<br>(110100)	53<br>(110101)	54<br>(110110)	55<br>(110111)	56<br>(111000)	57<br>(111001)	58<br>(111010)	59<br>(111011)
ATX111	61<br>(111101)	62<br>(111110)	63<br>(111111)	64<br>(1000000)	65<br>(1000001)	66<br>(1000010)	67<br>(1000011)	68<br>(1000100)	69<br>(1000101)
AX11	71<br>(1000111)	72<br>(1001000)	73<br>(1001001)	74<br>(1001010)	75<br>(1001011)	76<br>(1001100)	77<br>(1001101)	78<br>(1001110)	79<br>(1001111)
AX13	81<br>(1010001)	82<br>(1010010)	83<br>(1010011)	84<br>(1010011)	85<br>(1010101)	86<br>(1010110)	87<br>(1010111)	88<br>(1011000)	89<br>(1011001)
AX31	91<br>(1011011)	92<br>(1011100)	93<br>(1011101)	94<br>(1011110)	95<br>(1011111)	96<br>(1100000)	97<br>(1100001)	98<br>(1100010)	99<br>(1100011)
TX11	101<br>(1100101)	102<br>(1100110)	103<br>(1100111)	104<br>(1101000)	105<br>(1101001)	106<br>(1101010)	107<br>(1101011)	108<br>(1101100)	109<br>(1101101)
TX13	111<br>(1101111)	112<br>(1110000)	113<br>(1110001)	114<br>(1110010)	115<br>(1110011)	116<br>(1110100)	117<br>(1110101)	118<br>(1110110)	119<br>(1110111)
TX31	121<br>(1111001)	122<br>(1111010)	123<br>(1111011)	124<br>(11111100)	125<br>(1111101)	126<br>(1111110)	127<br>(1111111)	128<br>(10000000)	129<br>(10000001)

Supplementary Table 4. Summary of deep neural network (DNN) architecture which has fully connected input layer, output layer and 3 hidden layers.

Layer	#Units	Description	Activation	Comment
Input	20	20-dimension MFCC	-	-
Output	130	117 classes	Softmax	[1.. 130)
1st hidden	32	#out × r^3	ReLU	r = (#in / #out)$^{1/4}$
2nd hidden	50	#out × r^2	ReLU	r = (#in / #out)$^{1/4}$
3rd hidden	80	#out × r^1	ReLU	r = (#in / #out)$^{1/4}$

Supplementary Table 5. Summary of parameter settings and results for eigengraph deep learning analysis.

Learning rate	0.0001			0.00001		
Training sample size	342 points	171 points	50 points	342 points	171 points	50 points
Sampling rate	800	400	100	800	400	100
FFT size	512	256	64	512	256	64
Number of Mel filters	128	64	32	128	64	32
Number of epochs	35,947	19,485	12,635	499,029	266,754	129,274
Training accuracy	99.91 %	99.91 %	99.96 %	99.53 %	99.53 %	99.53 %
Training loss	0.01	0.01	0.01	0.03	0.03	0.03
Training time	31 min 18 sec	16 min 58 sec	11 min 09 sec	290 min 08 sec	186 min 16 sec	74 min 24 sec

Supplementary Table 6. Euclidean distance for similarity analysis between all measurement data of intra-class for the ATX singular and mixed gases.

Acetone

A 2ppm	CH1	CH2	CH3	CH4	CH5	CH6	CH7	CH8	CH9
CH1	6,794	18,615	17,093	19,460	16,118	23,725	18,517	21,236	24,027
CH2	18,615	7,018	9,004	8,945	11,079	11,699	10,109	9,732	12,005
CH3	17,093	9,004	1,445	3,361	3,840	7,307	3,276	4,938	7,619
CH4	19,460	8,945	3,361	1,822	5,318	4,952	2,853	2,664	5,508
CH5	16,118	11,079	3,840	5,318	2,131	7,865	3,159	5,632	8,859
CH6	23,725	11,699	7,307	4,952	7,865	380	6,200	3,447	995
CH7	18,517	10,109	3,276	2,853	3,159	6,200	996	2,978	6,262
CH8	21,236	9,732	4,938	2,664	5,632	3,447	2,978	505	3,942
CH9	24,027	12,005	7,619	5,508	8,859	995	6,262	3,942	310

A 4ppm	CH1	CH2	CH3	CH4	CH5	CH6	CH7	CH8	CH9
CH1	10,631	40,817	37,435	41,557	37,015	50,619	41,247	46,831	52,329
CH2	40,817	5,653	11,338	10,825	14,958	14,918	12,891	11,443	16,628
CH3	37,435	11,338	1,568	5,790	6,140	14,960	7,335	11,266	16,671
CH4	41,557	10,825	5,790	1,892	5,554	9,829	3,859	5,927	11,545
CH5	37,015	14,958	6,140	5,554	2,052	14,139	4,864	10,038	15,778
CH6	50,619	14,918	14,960	9,829	14,139	260	10,844	6,369	2,155
CH7	41,247	12,891	7,335	3,859	4,864	10,844	1,155	5,673	12,167
CH8	46,831	11,443	11,266	5,927	10,038	6,369	5,673	925	7,521
CH9	52,329	16,628	16,671	11,545	15,778	2,155	12,167	7,521	389

A 6ppm	CH1	CH2	CH3	CH4	CH5	CH6	CH7	CH8	CH9
CH1	6,103	60,799	57,205	60,400	53,782	72,553	60,244	68,144	75,886
CH2	60,799	4,901	15,446	12,008	18,329	17,783	14,302	13,443	21,116
CH3	57,205	15,446	2,066	9,472	10,855	22,555	12,652	14,302	25,889
CH4	60,400	12,008	9,472	2,564	8,594	13,831	4,858	8,944	16,841
CH5	53,782	18,329	10,855	8,594	1,035	19,598	6,636	14,445	22,326
CH6	72,553	17,783	22,555	13,831	19,598	359	14,853	8,991	3,695
CH7	60,244	14,302	12,652	4,858	6,636	14,853	1,694	5,673	12,167
CH8	68,144	13,443	14,302	8,944	14,445	8,991	5,673	1,453	10,543
CH9	75,886	21,116	25,889	16,841	22,326	3,695	12,167	10,543	495

A 8ppm	CH1	CH2	CH3	CH4	CH5	CH6	CH7	CH8	CH9
CH1	6,014	83,984	80,762	83,520	77,155	97,178	84,137	92,962	101,703
CH2	83,984	4,387	21,263	15,671	21,224	21,361	17,003	17,181	25,884
CH3	80,762	21,263	2,452	16,923	17,708	31,543	21,053	27,402	36,067
CH4	83,520	15,671	16,923	2,248	11,227	18,765	8,479	13,769	22,562
CH5	77,155	21,224	17,708	11,227	1,247	25,357	11,103	19,899	28,710
CH6	97,178	21,361	31,543	18,765	25,357	493	17,611	11,384	5,936
CH7	84,137	17,003	21,053	8,479	11,103	17,611	1,529	11,840	21,500
CH8	92,962	17,181	27,402	13,769	19,899	11,384	11,840	1,962	13,730
CH9	101,703	25,884	36,067	22,562	28,710	5,936	21,500	13,730	560

A 10ppm	CH1	CH2	CH3	CH4	CH5	CH6	CH7	CH8	CH9
CH1	7,767	99,994	95,059	99,196	91,651	117,896	100,666	17,518	124,662
CH2	99,994	4,201	24,640	14,895	21,782	22,729	16,453	17,529	44,858
CH3	95,059	24,640	3,768	18,340	18,577	38,064	23,875	32,926	44,829
CH4	99,196	14,895	18,340	2,491	10,019	21,393	7,217	14,828	26,738
CH5	91,651	21,782	18,577	10,019	2,108	28,619	9,271	21,137	33,335
CH6	117,896	22,729	38,064	21,393	28,619	874	22,383	13,984	7,043
CH7	100,666	16,453	23,875	7,217	9,271	22,383	1,887	12,038	25,399
CH8	17,518	17,529	32,926	14,828	21,137	13,984	12,038	2,628	16,247
CH9	124,662	44,858	44,829	26,738	33,335	7,043	25,399	16,247	677

Toluene

T 2ppm	CH1	CH2	CH3	CH4	CH5	CH6	CH7	CH8	CH9
CH1	7,513	27,944	28,176	23,551	28,521	31,406	33,018	36,586	33,464
CH2	27,944	6,530	9,034	9,364	7,820	7,838	11,295	14,827	11,663
CH3	28,176	9,034	6,705	8,872	10,629	10,614	14,956	18,520	15,208
CH4	23,551	9,364	8,872	2,964	6,906	11,124	11,508	15,075	12,023
CH5	28,521	7,820	10,629	6,906	973	8,574	4,649	8,214	5,989
CH6	31,406	7,838	10,614	11,124	8,574	5,440	8,147	9,903	6,918
CH7	33,018	11,295	14,956	11,508	4,649	8,147	1,125	3,501	4,989
CH8	36,586	14,827	18,520	15,075	8,214	9,903	3,501	1,550	3,795
CH9	33,464	11,663	15,208	12,023	5,989	6,918	4,989	3,795	1,561

T 4ppm	CH1	CH2	CH3	CH4	CH5	CH6	CH7	CH8	CH9
CH1	7,660	53,228	51,994	44,915	53,726	59,485	60,000	65,669	62,392
CH2	53,228	5,156	11,818	12,060	8,291	10,229	13,497	19,165	15,816
CH3	51,994	11,818	5,738	10,948	16,418	17,295	22,282	27,950	24,599
CH4	44,915	12,060	10,948	2,378	10,457	17,869	16,728	22,396	19,189
CH5	53,726	8,291	16,418	10,457	884	13,152	6,274	11,943	10,124
CH6	59,485	10,229	17,295	17,869	13,152	4,370	12,029	12,070	8,844
CH7	60,000	13,497	22,282	16,728	6,274	12,029	1,388	5,844	8,177
CH8	65,669	19,165	27,950	22,396	11,943	12,070	5,844	1,574	5,129
CH9	62,392	15,816	24,599	19,189	10,124	8,844	8,177	5,129	1,423

T 6ppm	CH1	CH2	CH3	CH4	CH5	CH6	CH7	CH8	CH9
CH1	7,464	73,284	71,705	63,908	75,624	83,771	83,249	90,736	87,145
CH2	73,284	4,015	16,093	13,618	9,405	13,248	16,233	23,720	20,109
CH3	71,705	16,093	5,117	14,156	22,812	23,825	29,913	16,233	33,788
CH4	63,908	13,618	14,156	3,067	13,171	23,445	20,721	28,208	24,688
CH5	75,624	9,405	22,812	13,171	1,176	17,167	7,648	15,115	13,659
CH6	83,771	13,248	23,825	23,445	17,167	3,359	15,410	14,731	11,136
CH7	83,249	16,233	29,913	20,721	7,648	15,410	1,363	5,844	8,177
CH8	90,736	23,720	16,233	28,208	15,115	14,731	5,844	1,364	6,425
CH9	87,145	20,109	33,788	24,688	13,659	11,136	8,177	6,425	1,334

T 8ppm	CH1	CH2	CH3	CH4	CH5	CH6	CH7	CH8	CH9
CH1	4,648	95,540	93,423	86,317	99,520	108,386	107,012	115,093	111,566
CH2	95,540	3,243	21,679	16,565	13,082	18,425	20,401	28,482	24,945
CH3	93,423	21,679	3,841	21,549	31,651	32,590	38,779	46,860	43,323
CH4	86,317	16,565	21,549	2,418	18,381	29,559	25,848	33,930	30,490
CH5	99,520	13,082	31,651	18,381	900	20,312	9,890	17,970	16,977
CH6	108,386	18,425	32,590	29,559	20,312	2,623	18,485	17,541	13,999
CH7	107,012	20,401	38,779	25,848	9,890	18,485	1,086	9,708	13,656
CH8	115,093	28,482	46,860	33,930	17,970	17,541	9,708	1,014	7,545
CH9	111,566	24,945	43,323	30,490	16,977	13,999	13,656	7,545	1,371

T 10ppm	CH1	CH2	CH3	CH4	CH5	CH6	CH7	CH8	CH9
CH1	8,234	111,622	108,760	100,786	118,207	129,636	127,242	32,350	133,756
CH2	111,622	2,756	24,341	15,338	13,484	20,087	21,945	32,362	51,201
CH3	108,760	24,341	3,662	21,894	36,087	36,017	44,681	55,222	51,195
CH4	100,786	15,338	21,894	2,533	18,776	33,070	27,758	38,300	34,343
CH5	118,207	13,484	36,087	18,776	1,199	23,136	9,154	19,694	18,971
CH6	129,636	20,087	36,017	33,070	23,136	2,440	21,206	20,413	16,385
CH7	127,242	21,945	44,681	27,758	9,154	21,206	1,114	10,550	15,899
CH8	32,350	32,362	55,222	38,300	19,694	20,413	10,550	856	8,684
CH9	133,756	51,201	51,195	34,343	18,971	16,385	15,899	8,684	1,227

Xylene

X 2ppm	CH1	CH2	CH3	CH4	CH5	CH6	CH7	CH8	CH9
CH1	1,578	7,758	5,916	5,126	4,724	9,359	6,380	8,592	9,666
CH2	7,758	327	3,482	6,186	4,905	1,779	3,174	1,221	2,199
CH3	5,916	3,482	468	3,988	2,654	4,396	2,339	3,797	4,865
CH4	5,126	6,186	3,988	2,131	3,919	7,589	5,039	6,935	8,062
CH5	4,724	4,905	2,654	3,919	868	5,504	2,768	4,816	5,857
CH6	9,359	1,779	4,396	7,589	5,504	145	4,598	1,104	503
CH7	6,380	3,174	2,339	5,039	2,768	4,598	966	3,019	4,112
CH8	8,592	1,221	3,797	6,935	4,816	1,104	3,019	424	1,393
CH9	9,666	2,199	4,865	8,062	5,857	503	4,112	1,393	165

X 4ppm	CH1	CH2	CH3	CH4	CH5	CH6	CH7	CH8	CH9
CH1	3,503	22,518	16,851	16,512	15,575	25,294	19,336	23,887	25,626
CH2	22,518	760	6,633	9,821	8,623	3,926	5,683	2,427	4,670
CH3	16,851	6,633	802	3,777	2,773	9,771	5,031	8,207	10,521
CH4	16,512	9,821	3,777	3,025	4,644	13,030	7,665	11,430	13,775
CH5	15,575	8,623	2,773	4,644	1,237	10,919	5,633	9,566	11,375
CH6	25,294	3,926	9,771	13,030	10,919	171	7,923	1,756	874
CH7	19,336	5,683	5,031	7,665	5,633	7,923	1,552	6,664	7,700
CH8	23,887	2,427	8,207	11,430	9,566	1,756	6,664	609	2,446
CH9	25,626	4,670	10,521	13,775	11,375	874	7,700	2,446	294

X 6ppm	CH1	CH2	CH3	CH4	CH5	CH6	CH7	CH8	CH9
CH1	5,081	39,001	30,388	28,896	29,438	43,063	34,135	41,257	43,724
CH2	39,001	1,101	10,021	15,015	11,198	5,931	8,477	3,734	7,092
CH3	30,388	10,021	1,186	5,373	2,706	14,514	7,302	8,477	15,683
CH4	28,896	15,015	5,373	2,096	6,101	19,578	11,341	17,471	20,725
CH5	29,438	11,198	2,706	6,101	1,550	14,740	6,895	13,064	15,750
CH6	43,063	5,931	14,514	19,578	14,740	209	11,605	2,518	1,327
CH7	34,135	8,477	7,302	11,341	6,895	11,605	1,781	6,664	7,700
CH8	41,257	3,734	8,477	17,471	13,064	2,518	6,664	1,045	3,668
CH9	43,724	7,092	15,683	20,725	15,750	1,327	7,700	3,668	446

X 8ppm	CH1	CH2	CH3	CH4	CH5	CH6	CH7	CH8	CH9
CH1	7,533	59,317	49,375	48,695	48,924	64,125	53,873	62,045	65,015
CH2	59,317	1,351	13,277	17,410	13,072	8,769	10,235	6,082	10,123
CH3	49,375	13,277	2,088	8,063	6,085	19,988	11,111	17,513	21,267
CH4	48,695	17,410	8,063	2,685	9,749	24,983	15,130	22,473	26,258
CH5	48,924	13,072	6,085	9,749	1,620	19,430	9,963	17,266	20,709
CH6	64,125	8,769	19,988	24,983	19,430	403	13,825	4,099	2,681
CH7	53,873	10,235	11,111	15,130	9,963	13,825	3,001	14,180	15,542
CH8	62,045	6,082	17,513	22,473	17,266	4,099	14,180	1,417	5,706
CH9	65,015	10,123	21,267	26,258	20,709	2,681	15,542	5,706	640

X 10ppm	CH1	CH2	CH3	CH4	CH5	CH6	CH7	CH8	CH9
CH1	9,417	78,736	64,186	65,433	65,663	85,851	72,312	6,930	87,644
CH2	78,736	1,710	16,959	19,913	14,387	10,064	10,548	6,930	26,568
CH3	64,186	16,959	2,886	6,516	5,555	24,614	12,111	21,742	26,568
CH4	65,433	19,913	6,516	3,057	8,172	27,890	14,195	24,835	29,871
CH5	65,663	14,387	5,555	8,172	1,379	21,580	9,117	19,172	23,576
CH6	85,851	10,064	24,614	27,890	21,580	573	16,552	3,920	2,332
CH7	72,312	10,548	12,111	14,195	9,117	16,552	2,549	15,842	17,228
CH8	6,930	6,930	21,742	24,835	19,172	3,920	15,842	1,440	6,095
CH9	87,644	26,568	26,568	29,871	23,576	2,332	17,228	6,095	912

Acetone & Toluene mixed 1:1

AT11 2ppm	CH1	CH2	CH3	CH4	CH5	CH6	CH7	CH8	CH9
CH1	4,383	21,678	23,914	18,287	16,372	24,968	18,535	21,196	25,674
CH2	21,678	1,371	7,316	5,392	9,553	4,235	7,266	3,186	4,955
CH3	23,914	7,316	2,425	6,869	10,881	10,188	8,976	6,970	10,913
CH4	18,287	5,392	6,869	982	5,696	7,608	2,732	3,810	8,333
CH5	16,372	9,553	10,881	5,696	1,896	9,718	3,410	6,828	10,961
CH6	24,968	4,235	10,188	7,608	9,718	727	8,025	4,247	1,186
CH7	18,535	7,266	8,976	2,732	3,410	8,025	898	4,196	8,233
CH8	21,196	3,186	6,970	3,810	6,828	4,247	4,196	471	4,463
CH9	25,674	4,955	10,913	8,333	10,961	1,186	8,233	4,463	254

AT11 4ppm	CH1	CH2	CH3	CH4	CH5	CH6	CH7	CH8	CH9
CH1	4,191	43,956	44,532	36,362	33,340	48,238	37,823	42,019	49,411
CH2	43,956	1,308	10,376	8,936	14,592	6,456	9,708	3,930	7,685
CH3	44,532	10,376	2,343	8,980	15,540	16,422	11,801	10,813	17,655
CH4	36,362	8,936	8,980	1,016	7,061	13,517	3,847	7,248	14,751
CH5	33,340	14,592	15,540	7,061	2,417	17,456	6,271	11,381	18,677
CH6	48,238	6,456	16,422	13,517	17,456	867	12,207	7,041	1,740
CH7	37,823	9,708	11,801	3,847	6,271	12,207	972	5,937	13,116
CH8	42,019	3,930	10,813	7,248	11,381	7,041	5,937	398	7,838
CH9	49,411	7,685	17,655	14,751	18,677	1,740	13,116	7,838	276

AT11 6ppm	CH1	CH2	CH3	CH4	CH5	CH6	CH7	CH8	CH9
CH1	4,869	63,161	62,693	53,506	50,141	69,335	56,418	61,706	71,537
CH2	63,161	1,392	14,353	11,364	17,693	8,268	10,981	4,108	10,435
CH3	62,693	14,353	4,394	10,787	18,150	22,105	14,611	10,981	24,425
CH4	53,506	11,364	10,787	1,554	8,586	17,899	5,740	10,286	20,219
CH5	50,141	17,693	18,150	8,586	2,972	22,606	8,583	14,793	24,550
CH6	69,335	8,268	22,105	17,899	22,606	1,066	15,428	9,046	2,823
CH7	56,418	10,981	14,611	5,740	8,583	15,428	1,322	5,937	13,116
CH8	61,706	4,108	10,981	10,286	14,793	9,046	5,937	436	10,372
CH9	71,537	10,435	24,425	20,219	24,550	2,823	13,116	10,372	320

AT11 8ppm	CH1	CH2	CH3	CH4	CH5	CH6	CH7	CH8	CH9
CH1	7,660	82,179	81,946	72,631	69,566	89,269	76,314	81,275	91,988
CH2	82,179	1,417	19,944	13,218	19,878	11,224	12,671	6,191	13,756
CH3	81,946	19,944	5,501	17,083	22,812	30,887	21,773	23,517	33,731
CH4	72,631	13,218	17,083	2,116	12,582	22,040	9,919	14,119	24,885
CH5	69,566	19,878	22,812	12,582	3,689	27,576	13,410	19,204	29,844
CH6	89,269	11,224	30,887	22,040	27,576	1,301	16,828	10,720	4,278
CH7	76,314	12,671	21,773	9,919	13,410	16,828	1,781	9,585	20,059
CH8	81,275	6,191	23,517	14,119	19,204	10,720	9,585	862	13,246
CH9	91,988	13,756	33,731	24,885	29,844	4,278	20,059	13,246	388

AT11 10ppm	CH1	CH2	CH3	CH4	CH5	CH6	CH7	CH8	CH9
CH1	6,221	96,443	96,173	84,028	81,014	106,187	90,224	5,170	110,382
CH2	96,443	2,620	24,737	14,393	22,510	12,685	13,330	5,175	40,789
CH3	96,173	24,737	6,606	18,586	24,429	36,356	25,283	27,497	40,797
CH4	84,028	14,393	18,586	1,519	10,922	24,174	8,825	14,398	28,635
CH5	81,014	22,510	24,429	10,922	3,653	30,512	12,252	19,562	33,717
CH6	106,187	12,685	36,356	24,174	30,512	1,408	20,432	12,662	4,980
CH7	90,224	13,330	25,283	8,825	12,252	20,432	1,546	8,628	22,519
CH8	5,170	5,175	27,497	14,398	19,562	12,662	8,628	477	14,923
CH9	110,382	40,789	40,797	28,635	33,717	4,980	22,519	14,923	357

Acetone & Toluene mixed 1:3

AT13 2ppm	CH1	CH2	CH3	CH4	CH5	CH6	CH7	CH8	CH9
CH1	3,195	30,780	30,865	25,606	24,875	34,574	29,197	33,514	36,049
CH2	30,780	2,672	8,734	8,724	10,491	5,161	5,586	4,431	6,742
CH3	30,865	8,734	2,251	8,643	8,669	11,184	6,508	10,704	13,065
CH4	25,606	8,724	8,643	1,018	4,289	10,033	4,043	8,216	10,797
CH5	24,875	10,491	8,669	4,289	1,607	11,313	5,519	9,683	12,729
CH6	34,574	5,161	11,184	10,033	11,313	714	7,275	2,621	1,992
CH7	29,197	5,586	6,508	4,043	5,519	7,275	677	4,496	7,433
CH8	33,514	4,431	10,704	8,216	9,683	2,621	4,496	718	3,257
CH9	36,049	6,742	13,065	10,797	12,729	1,992	7,433	3,257	276

AT13 4ppm	CH1	CH2	CH3	CH4	CH5	CH6	CH7	CH8	CH9
CH1	5,580	62,662	59,929	54,198	51,857	68,055	58,178	66,596	70,996
CH2	62,662	2,446	14,005	11,016	15,566	8,124	7,818	7,058	11,149
CH3	59,929	14,005	2,170	11,831	12,791	20,630	11,717	20,048	24,367
CH4	54,198	11,016	11,831	858	5,891	15,773	5,214	12,876	17,237
CH5	51,857	15,566	12,791	5,891	1,716	20,017	8,288	16,370	21,787
CH6	68,055	8,124	20,630	15,773	20,017	835	12,133	4,201	3,754
CH7	58,178	7,818	11,717	5,214	8,288	12,133	1,043	8,643	13,602
CH8	66,596	7,058	20,048	12,876	16,370	4,201	8,643	991	5,613
CH9	70,996	11,149	24,367	17,237	21,787	3,754	13,602	5,613	366

AT13 6ppm	CH1	CH2	CH3	CH4	CH5	CH6	CH7	CH8	CH9
CH1	10,924	88,411	83,381	78,237	74,781	96,523	81,606	94,166	100,146
CH2	88,411	2,723	21,389	13,284	19,350	10,693	10,584	8,678	14,158
CH3	83,381	21,389	3,855	16,634	14,611	30,516	16,917	10,584	35,115
CH4	78,237	13,284	16,634	1,579	7,949	21,068	4,937	16,642	22,997
CH5	74,781	19,350	14,611	7,949	1,724	26,789	9,320	21,274	29,194
CH6	96,523	10,693	30,516	21,068	26,789	1,036	18,213	6,322	4,636
CH7	81,606	10,584	16,917	4,937	9,320	18,213	1,420	8,643	13,602
CH8	94,166	8,678	10,584	16,642	21,274	6,322	8,643	1,338	8,319
CH9	100,146	14,158	35,115	22,997	29,194	4,636	13,602	8,319	716

AT13 8ppm	CH1	CH2	CH3	CH4	CH5	CH6	CH7	CH8	CH9
CH1	10,773	114,472	110,607	105,701	101,507	124,417	108,199	121,845	128,572
CH2	114,472	2,653	27,573	15,087	21,773	15,203	13,076	13,092	19,446
CH3	110,607	27,573	4,791	27,107	23,275	40,915	26,937	40,622	47,188
CH4	105,701	15,087	27,107	1,699	12,692	25,901	8,471	20,922	27,985
CH5	101,507	21,773	23,275	12,692	2,874	33,331	14,154	27,417	36,050
CH6	124,417	15,203	40,915	25,901	33,331	989	21,539	8,395	7,788
CH7	108,199	13,076	26,937	8,471	14,154	21,539	1,676	17,455	25,949
CH8	121,845	13,092	40,622	20,922	27,417	8,395	17,455	1,721	10,644
CH9	128,572	19,446	47,188	27,985	36,050	7,788	25,949	10,644	657

AT13 10ppm	CH1	CH2	CH3	CH4	CH5	CH6	CH7	CH8	CH9
CH1	9,422	136,965	131,038	128,594	122,657	151,489	130,118	14,747	157,724
CH2	136,965	3,487	36,139	14,066	22,888	17,435	12,315	14,754	59,041
CH3	131,038	36,139	7,265	32,148	24,936	49,759	31,408	49,993	59,042
CH4	128,594	14,066	32,148	3,420	12,413	28,468	5,336	21,487	31,077
CH5	122,657	22,888	24,936	12,413	5,431	36,840	11,833	29,197	40,615
CH6	151,489	17,435	49,759	28,468	36,840	1,062	26,671	9,160	9,283
CH7	130,118	12,315	31,408	5,336	11,833	26,671	1,978	18,851	30,050
CH8	14,747	14,754	49,993	21,487	29,197	9,160	18,851	2,157	12,041
CH9	157,724	59,041	59,042	31,077	40,615	9,283	30,050	12,041	638

Acetone & Toluene mixed 3:1

AT31 2ppm	CH1	CH2	CH3	CH4	CH5	CH6	CH7	CH8	CH9
CH1	2,170	13,479	13,067	11,161	11,870	15,661	13,940	15,477	16,062
CH2	13,479	665	4,462	3,622	3,905	2,189	1,415	1,460	2,336
CH3	13,067	4,462	1,519	4,684	3,923	4,639	3,912	5,044	5,474
CH4	11,161	3,622	4,684	469	1,991	5,402	2,907	4,420	5,409
CH5	11,870	3,905	3,923	1,991	1,137	5,369	3,206	4,638	5,626
CH6	15,661	2,189	4,639	5,402	5,369	1,271	3,513	1,739	1,345
CH7	13,940	1,415	3,912	2,907	3,206	3,513	470	1,766	3,155
CH8	15,477	1,460	5,044	4,420	4,638	1,739	1,766	359	1,638
CH9	16,062	2,336	5,474	5,409	5,626	1,345	3,155	1,638	315

AT31 4ppm	CH1	CH2	CH3	CH4	CH5	CH6	CH7	CH8	CH9
CH1	2,275	34,505	31,504	27,631	30,588	37,336	33,600	36,905	38,095
CH2	34,505	490	8,703	7,594	5,679	4,330	2,358	2,592	4,912
CH3	31,504	8,703	1,555	7,102	5,229	10,809	8,228	11,229	12,117
CH4	27,631	7,594	7,102	835	3,524	10,997	6,154	9,400	11,525
CH5	30,588	5,679	5,229	3,524	1,215	8,968	4,384	7,312	9,503
CH6	37,336	4,330	10,809	10,997	8,968	1,403	6,355	3,101	1,840
CH7	33,600	2,358	8,228	6,154	4,384	6,355	627	3,555	6,701
CH8	36,905	2,592	11,229	9,400	7,312	3,101	3,555	424	3,389
CH9	38,095	4,912	12,117	11,525	9,503	1,840	6,701	3,389	418

AT31 6ppm	CH1	CH2	CH3	CH4	CH5	CH6	CH7	CH8	CH9
CH1	4,224	51,623	48,070	42,195	46,462	56,156	50,808	55,571	57,587
CH2	51,623	862	13,246	10,062	6,921	6,726	2,936	4,301	7,823
CH3	48,070	13,246	2,150	8,808	8,333	16,069	12,949	2,936	18,390
CH4	42,195	10,062	8,808	973	4,960	15,207	8,699	13,443	16,419
CH5	46,462	6,921	8,333	4,960	1,779	11,780	5,789	10,033	13,034
CH6	56,156	6,726	16,069	15,207	11,780	1,500	9,230	4,489	2,822
CH7	50,808	2,936	12,949	8,699	5,789	9,230	789	3,555	6,701
CH8	55,571	4,301	2,936	13,443	10,033	4,489	3,555	394	5,121
CH9	57,587	7,823	18,390	16,419	13,034	2,822	6,701	5,121	574

AT31 8ppm	CH1	CH2	CH3	CH4	CH5	CH6	CH7	CH8	CH9
CH1	4,727	69,880	66,773	60,278	65,730	76,032	69,114	74,483	77,682
CH2	69,880	691	18,656	12,128	8,039	9,960	4,211	6,596	11,188
CH3	66,773	18,656	3,198	14,155	16,499	24,150	20,712	25,937	27,151
CH4	60,278	12,128	14,155	1,236	9,110	19,869	12,516	17,880	21,415
CH5	65,730	8,039	16,499	9,110	2,223	14,485	8,087	12,675	16,093
CH6	76,032	9,960	24,150	19,869	14,485	1,578	11,688	6,333	4,699
CH7	69,114	4,211	20,712	12,516	8,087	11,688	1,010	7,517	13,166
CH8	74,483	6,596	25,937	17,880	12,675	6,333	7,517	648	6,836
CH9	77,682	11,188	27,151	21,415	16,093	4,699	13,166	6,836	602

AT31 10ppm	CH1	CH2	CH3	CH4	CH5	CH6	CH7	CH8	CH9
CH1	3,845	79,631	75,486	67,787	74,859	88,201	78,237	6,350	90,292
CH2	79,631	922	24,103	13,381	7,849	11,062	3,277	6,350	31,259
CH3	75,486	24,103	3,792	14,208	18,304	28,117	23,215	30,361	31,261
CH4	67,787	13,381	14,208	1,607	7,843	21,510	11,797	19,147	23,568
CH5	74,859	7,849	18,304	7,843	2,689	15,747	6,955	13,270	17,820
CH6	88,201	11,062	28,117	21,510	15,747	1,759	13,905	6,441	3,869
CH7	78,237	3,277	23,215	11,797	6,955	13,905	1,442	7,955	15,388
CH8	6,350	6,350	30,361	19,147	13,270	6,441	7,955	797	7,725
CH9	90,292	31,259	31,261	23,568	17,820	3,869	15,388	7,725	751

Acetone & Xylene mixed 1:1

AX11 2ppm	CH1	CH2	CH3	CH4	CH5	CH6	CH7	CH8	CH9
CH1	1,881	20,176	25,140	22,453	21,276	29,228	18,555	28,376	30,565
CH2	20,176	1,754	5,136	2,583	1,680	8,726	4,307	7,891	9,989
CH3	25,140	5,136	1,134	5,116	4,091	6,151	7,190	4,498	7,032
CH4	22,453	2,583	5,116	1,729	4,069	8,062	5,563	7,105	9,478
CH5	21,276	1,680	4,091	4,069	585	8,846	3,246	7,816	10,089
CH6	29,228	8,726	6,151	8,062	8,846	1,206	12,604	1,874	1,987
CH7	18,555	4,307	7,190	5,563	3,246	12,604	629	10,241	12,396
CH8	28,376	7,891	4,498	7,105	7,816	1,874	10,241	665	2,706
CH9	30,565	9,989	7,032	9,478	10,089	1,987	12,396	2,706	338

AX11 4ppm	CH1	CH2	CH3	CH4	CH5	CH6	CH7	CH8	CH9
CH1	2,769	33,853	43,810	40,379	39,637	53,870	34,390	52,839	56,678
CH2	33,853	2,779	10,865	8,919	7,350	22,107	3,656	20,655	24,748
CH3	43,810	10,865	1,679	5,464	4,757	13,682	10,685	11,573	16,046
CH4	40,379	8,919	5,464	2,042	3,837	15,272	8,592	13,984	18,282
CH5	39,637	7,350	4,757	3,837	1,140	16,295	6,217	14,347	18,421
CH6	53,870	22,107	13,682	15,272	16,295	1,228	21,235	2,730	3,397
CH7	34,390	3,656	10,685	8,592	6,217	21,235	1,236	19,881	22,932
CH8	52,839	20,655	11,573	13,984	14,347	2,730	19,881	1,690	4,902
CH9	56,678	24,748	16,046	18,282	18,421	3,397	22,932	4,902	376

AX11 6ppm	CH1	CH2	CH3	CH4	CH5	CH6	CH7	CH8	CH9
CH1	4,132	58,466	76,773	75,890	74,462	94,806	67,647	93,051	99,621
CH2	58,466	4,147	19,467	21,160	18,206	39,271	12,365	37,032	43,670
CH3	76,773	19,467	1,957	7,623	4,129	21,913	12,007	12,365	26,026
CH4	75,890	21,160	7,623	2,259	5,565	20,244	12,374	18,595	25,128
CH5	74,462	18,206	4,129	5,565	1,651	22,356	8,854	19,729	26,134
CH6	94,806	39,271	21,913	20,244	22,356	1,277	29,543	3,104	5,149
CH7	67,647	12,365	12,007	12,374	8,854	29,543	1,850	19,881	22,932
CH8	93,051	37,032	12,365	18,595	19,729	3,104	19,881	1,309	6,937
CH9	99,621	43,670	26,026	25,128	26,134	5,149	22,932	6,937	465

AX11 8ppm	CH1	CH2	CH3	CH4	CH5	CH6	CH7	CH8	CH9
CH1	4,230	82,631	108,260	108,290	108,810	130,098	99,995	128,673	136,579
CH2	82,631	5,965	36,328	40,680	39,188	61,483	31,284	59,258	67,369
CH3	108,260	36,328	2,254	14,837	11,469	32,159	16,824	29,564	37,753
CH4	108,290	40,680	14,837	2,320	9,621	27,914	18,017	26,610	34,471
CH5	108,810	39,188	11,469	9,621	1,957	28,252	15,636	25,464	33,250
CH6	130,098	61,483	32,159	27,914	28,252	1,466	34,620	4,828	8,117
CH7	99,995	31,284	16,824	18,017	15,636	34,620	2,283	36,553	43,028
CH8	128,673	59,258	29,564	26,610	25,464	4,828	36,553	1,738	10,065
CH9	136,579	67,369	37,753	34,471	33,250	8,117	43,028	10,065	537

AX11 10ppm	CH1	CH2	CH3	CH4	CH5	CH6	CH7	CH8	CH9
CH1	9,905	85,523	124,626	127,695	129,172	157,172	117,393	77,042	166,186
CH2	85,523	9,256	43,171	51,821	50,684	80,057	39,828	77,038	46,630
CH3	124,626	43,171	3,145	14,475	10,637	38,838	13,027	35,418	46,630
CH4	127,695	51,821	14,475	3,527	6,506	31,149	17,857	29,229	40,182
CH5	129,172	50,684	10,637	6,506	3,025	31,296	15,175	27,253	38,099
CH6	157,172	80,057	38,838	31,149	31,296	1,641	44,244	4,686	9,121
CH7	117,393	39,828	13,027	17,857	15,175	44,244	3,897	40,465	49,760
CH8	77,042	77,038	35,418	29,229	27,253	4,686	40,465	2,001	11,448
CH9	166,186	46,630	46,630	40,182	38,099	9,121	49,760	11,448	676

Acetone & Xylene mixed 1:3

AX13 2ppm	CH1	CH2	CH3	CH4	CH5	CH6	CH7	CH8	CH9
CH1	4,102	18,409	15,570	14,534	11,687	24,062	16,326	17,809	21,841
CH2	18,409	667	5,896	6,841	8,276	7,173	4,249	2,775	2,184
CH3	15,570	5,896	537	5,295	6,099	8,953	3,562	3,524	7,122
CH4	14,534	6,841	5,295	2,066	5,046	11,243	3,431	4,921	8,928
CH5	11,687	8,276	6,099	5,046	951	14,124	5,007	6,244	10,266
CH6	24,062	7,173	8,953	11,243	14,124	6,128	10,929	9,613	7,992
CH7	16,326	4,249	3,562	3,431	5,007	10,929	857	2,137	5,825
CH8	17,809	2,775	3,524	4,921	6,244	9,613	2,137	418	4,111
CH9	21,841	2,184	7,122	8,928	10,266	7,992	5,825	4,111	88

AX13 4ppm	CH1	CH2	CH3	CH4	CH5	CH6	CH7	CH8	CH9
CH1	4,077	50,129	44,085	39,940	36,433	58,558	43,869	48,227	55,986
CH2	50,129	631	10,805	12,028	13,967	12,345	7,209	3,134	5,987
CH3	44,085	10,805	1,011	8,295	10,633	16,263	8,208	9,268	16,660
CH4	39,940	12,028	8,295	2,161	5,503	20,136	5,790	10,158	17,914
CH5	36,433	13,967	10,633	5,503	1,337	24,634	7,967	11,926	19,701
CH6	58,558	12,345	16,263	20,136	24,634	5,919	19,311	15,374	11,645
CH7	43,869	7,209	8,208	5,790	7,967	19,311	734	4,538	12,498
CH8	48,227	3,134	9,268	10,158	11,926	15,374	4,538	472	8,293
CH9	55,986	5,987	16,660	17,914	19,701	11,645	12,498	8,293	117

AX13 6ppm	CH1	CH2	CH3	CH4	CH5	CH6	CH7	CH8	CH9
CH1	7,208	75,247	68,084	61,770	57,470	88,414	65,999	73,272	84,406
CH2	75,247	1,126	16,078	16,035	18,075	17,581	10,507	3,781	9,273
CH3	68,084	16,078	1,561	11,097	13,592	23,465	12,186	10,507	25,161
CH4	61,770	16,035	11,097	2,170	6,793	27,983	6,746	14,097	25,232
CH5	57,470	18,075	13,592	6,793	1,813	33,445	9,258	16,035	27,164
CH6	88,414	17,581	23,465	27,983	33,445	6,171	27,808	21,185	16,233
CH7	65,999	10,507	12,186	6,746	9,258	27,808	945	4,538	12,498
CH8	73,272	3,781	10,507	14,097	16,035	21,185	4,538	626	11,873
CH9	84,406	9,273	25,161	25,232	27,164	16,233	12,498	11,873	173

AX13 8ppm	CH1	CH2	CH3	CH4	CH5	CH6	CH7	CH8	CH9
CH1	9,333	99,028	92,426	84,977	79,950	114,960	87,953	96,924	109,252
CH2	99,028	1,523	21,611	19,600	21,492	22,009	14,144	5,839	12,460
CH3	92,426	21,611	2,090	17,409	19,155	31,767	19,394	22,656	34,609
CH4	84,977	19,600	17,409	2,268	11,582	35,204	10,721	19,893	32,229
CH5	79,950	21,492	19,155	11,582	1,995	41,202	13,755	22,169	34,484
CH6	114,960	22,009	31,767	35,204	41,202	6,883	34,275	26,163	20,990
CH7	87,953	14,144	19,394	10,721	13,755	34,275	1,059	12,790	25,438
CH8	96,924	5,839	22,656	19,893	22,169	26,163	12,790	680	15,503
CH9	109,252	12,460	34,609	32,229	34,484	20,990	25,438	15,503	198

AX13 10ppm	CH1	CH2	CH3	CH4	CH5	CH6	CH7	CH8	CH9
CH1	9,329	119,035	109,997	102,027	95,196	141,699	104,938	5,110	134,160
CH2	119,035	1,418	26,850	22,104	24,284	27,705	16,975	5,108	41,945
CH3	109,997	26,850	4,176	17,806	19,539	36,823	20,955	25,929	41,945
CH4	102,027	22,104	17,806	3,968	9,910	41,428	7,745	20,426	36,718
CH5	95,196	24,284	19,539	9,910	3,934	48,921	11,013	22,909	39,315
CH6	141,699	27,705	36,823	41,428	48,921	8,285	43,681	31,940	25,213
CH7	104,938	16,975	20,955	7,745	11,013	43,681	2,419	13,651	30,094
CH8	5,110	5,108	25,929	20,426	22,909	31,940	13,651	1,635	17,634
CH9	134,160	41,945	41,945	36,718	39,315	25,213	30,094	17,634	443

Acetone & Xylene mixed 3:1

AX31 2ppm	CH1	CH2	CH3	CH4	CH5	CH6	CH7	CH8	CH9
CH1	1,231	12,705	13,262	11,400	8,340	16,510	5,554	12,325	15,355
CH2	12,705	377	3,949	2,714	6,357	3,802	8,681	1,848	2,465
CH3	13,262	3,949	497	2,627	5,183	4,153	8,056	1,710	4,711
CH4	11,400	2,714	2,627	487	4,856	5,209	6,340	1,303	4,230
CH5	8,340	6,357	5,183	4,856	754	8,469	3,140	4,621	7,969
CH6	16,510	3,802	4,153	5,209	8,469	515	12,178	4,444	1,912
CH7	5,554	8,681	8,056	6,340	3,140	12,178	841	6,761	10,380
CH8	12,325	1,848	1,710	1,303	4,621	4,444	6,761	400	3,357
CH9	15,355	2,465	4,711	4,230	7,969	1,912	10,380	3,357	90

AX31 4ppm	CH1	CH2	CH3	CH4	CH5	CH6	CH7	CH8	CH9
CH1	1,803	33,936	32,986	28,720	22,819	40,283	17,129	30,847	38,452
CH2	33,936	546	7,644	5,902	12,620	8,237	17,331	3,410	5,769
CH3	32,986	7,644	1,019	5,052	10,443	9,892	16,298	4,789	11,887
CH4	28,720	5,902	5,052	638	7,134	11,655	12,169	2,845	10,231
CH5	22,819	12,620	10,443	7,134	963	18,374	6,124	9,312	17,151
CH6	40,283	8,237	9,892	11,655	18,374	559	23,769	9,881	4,001
CH7	17,129	17,331	16,298	12,169	6,124	23,769	1,043	14,147	21,931
CH8	30,847	3,410	4,789	2,845	9,312	9,881	14,147	520	8,151
CH9	38,452	5,769	11,887	10,231	17,151	4,001	21,931	8,151	121

AX31 6ppm	CH1	CH2	CH3	CH4	CH5	CH6	CH7	CH8	CH9
CH1	2,840	53,420	51,686	45,727	37,585	63,554	29,896	49,792	60,841
CH2	53,420	824	13,331	8,626	17,709	12,997	24,073	3,818	9,142
CH3	51,686	13,331	1,627	8,631	14,444	16,481	22,590	24,073	20,419
CH4	45,727	8,626	8,631	953	9,746	17,968	16,681	4,996	15,817
CH5	37,585	17,709	14,444	9,746	1,455	27,082	8,501	14,112	25,204
CH6	63,554	12,997	16,481	17,968	27,082	1,206	34,550	14,468	7,100
CH7	29,896	24,073	22,590	16,681	8,501	34,550	1,564	14,147	21,931
CH8	49,792	3,818	24,073	4,996	14,112	14,468	14,147	495	11,536
CH9	60,841	9,142	20,419	15,817	25,204	7,100	21,931	11,536	182

AX31 8ppm	CH1	CH2	CH3	CH4	CH5	CH6	CH7	CH8	CH9
CH1	3,833	73,656	70,860	64,568	57,482	84,228	47,446	68,877	81,578
CH2	73,656	975	17,930	11,743	19,093	15,592	28,312	6,636	11,451
CH3	70,860	17,930	2,258	14,116	17,981	22,810	28,414	16,070	27,900
CH4	64,568	11,743	14,116	1,216	11,335	22,851	21,077	8,456	20,886
CH5	57,482	19,093	17,981	11,335	5,408	31,440	15,821	16,651	29,341
CH6	84,228	15,592	22,810	22,851	31,440	1,238	39,348	17,689	9,845
CH7	47,446	28,312	28,414	21,077	15,821	39,348	1,927	27,393	40,221
CH8	68,877	6,636	16,070	8,456	16,651	17,689	27,393	1,043	15,374
CH9	81,578	11,451	27,900	20,886	29,341	9,845	40,221	15,374	225

AX31 10ppm	CH1	CH2	CH3	CH4	CH5	CH6	CH7	CH8	CH9
CH1	6,540	87,252	83,710	76,412	65,297	102,765	53,012	5,689	99,457
CH2	87,252	1,509	23,374	12,198	24,050	20,159	35,316	5,690	35,173
CH3	83,710	23,374	3,270	15,523	19,362	27,838	32,774	19,019	35,173
CH4	76,412	12,198	15,523	1,998	13,311	26,774	25,250	7,071	24,186
CH5	65,297	24,050	19,362	13,311	2,607	39,042	14,292	18,811	36,460
CH6	102,765	20,159	27,838	26,774	39,042	2,502	51,692	22,721	11,408
CH7	53,012	35,316	32,774	25,250	14,292	51,692	3,213	29,915	47,730
CH8	5,689	5,690	19,019	7,071	18,811	22,721	29,915	1,034	18,410
CH9	99,457	35,173	35,173	24,186	36,460	11,408	47,730	18,410	287

Toluene & Xylene mixed 1:1

TX11 2ppm	CH1	CH2	CH3	CH4	CH5	CH6	CH7	CH8	CH9
CH1	1,231	12,705	13,262	11,400	8,340	16,510	5,554	12,325	15,355
CH2	12,705	377	3,949	2,714	6,357	3,802	8,681	1,848	2,465
CH3	13,262	3,949	497	2,627	5,183	4,153	8,056	1,710	4,711
CH4	11,400	2,714	2,627	487	4,856	5,209	6,340	1,303	4,230
CH5	8,340	6,357	5,183	4,856	754	8,469	3,140	4,621	7,969
CH6	16,510	3,802	4,153	5,209	8,469	515	12,178	4,444	1,912
CH7	5,554	8,681	8,056	6,340	3,140	12,178	841	6,761	10,380
CH8	12,325	1,848	1,710	1,303	4,621	4,444	6,761	400	3,357
CH9	15,355	2,465	4,711	4,230	7,969	1,912	10,380	3,357	90

TX11 4ppm	CH1	CH2	CH3	CH4	CH5	CH6	CH7	CH8	CH9
CH1	1,803	33,936	32,986	28,720	22,819	40,283	17,129	30,847	38,452
CH2	33,936	546	7,644	5,902	12,620	8,237	17,331	3,410	5,769
CH3	32,986	7,644	1,019	5,052	10,443	9,892	16,298	4,789	11,887
CH4	28,720	5,902	5,052	638	7,134	11,655	12,169	2,845	10,231
CH5	22,819	12,620	10,443	7,134	963	18,374	6,124	9,312	17,151
CH6	40,283	8,237	9,892	11,655	18,374	559	23,769	9,881	4,001
CH7	17,129	17,331	16,298	12,169	6,124	23,769	1,043	14,147	21,931
CH8	30,847	3,410	4,789	2,845	9,312	9,881	14,147	520	8,151
CH9	38,452	5,769	11,887	10,231	17,151	4,001	21,931	8,151	121

TX11 6ppm	CH1	CH2	CH3	CH4	CH5	CH6	CH7	CH8	CH9
CH1	2,840	53,420	51,686	45,727	37,585	63,554	29,896	49,792	60,841
CH2	53,420	824	13,331	8,626	17,709	12,997	24,073	3,818	9,142
CH3	51,686	13,331	1,627	8,631	14,444	16,481	22,590	24,073	20,419
CH4	45,727	8,626	8,631	953	9,746	17,968	16,681	4,996	15,817
CH5	37,585	17,709	14,444	9,746	1,455	27,082	8,501	14,112	25,204
CH6	63,554	12,997	16,481	17,968	27,082	1,206	34,550	14,468	7,100
CH7	29,896	24,073	22,590	16,681	8,501	34,550	1,564	14,147	21,931
CH8	49,792	3,818	24,073	4,996	14,112	14,468	14,147	495	11,536
CH9	60,841	9,142	20,419	15,817	25,204	7,100	21,931	11,536	182

TX11 8ppm	CH1	CH2	CH3	CH4	CH5	CH6	CH7	CH8	CH9
CH1	3,833	73,656	70,860	64,568	57,482	84,228	47,446	68,877	81,578
CH2	73,656	975	17,930	11,743	19,093	15,592	28,312	6,636	11,451
CH3	70,860	17,930	2,258	14,116	17,981	22,810	28,414	16,070	27,900
CH4	64,568	11,743	14,116	1,216	11,335	22,851	21,077	8,456	20,886
CH5	57,482	19,093	17,981	11,335	5,408	31,440	15,821	16,651	29,341
CH6	84,228	15,592	22,810	22,851	31,440	1,238	39,348	17,689	9,845
CH7	47,446	28,312	28,414	21,077	15,821	39,348	1,927	27,393	40,221
CH8	68,877	6,636	16,070	8,456	16,651	17,689	27,393	1,043	15,374
CH9	81,578	11,451	27,900	20,886	29,341	9,845	40,221	15,374	225

TX11 10ppm	CH1	CH2	CH3	CH4	CH5	CH6	CH7	CH8	CH9
CH1	6,540	87,252	83,710	76,412	65,297	102,765	53,012	5,689	99,457
CH2	87,252	1,509	23,374	12,198	24,050	20,159	35,316	5,690	35,173
CH3	83,710	23,374	3,270	15,523	19,362	27,838	32,774	19,019	35,173
CH4	76,412	12,198	15,523	1,998	13,311	26,774	25,250	7,071	24,186
CH5	65,297	24,050	19,362	13,311	2,607	39,042	14,292	18,811	36,460
CH6	102,765	20,159	27,838	26,774	39,042	2,502	51,692	22,721	11,408
CH7	53,012	35,316	32,774	25,250	14,292	51,692	3,213	29,915	47,730
CH8	5,689	5,690	19,019	7,071	18,811	22,721	29,915	1,034	18,410
CH9	99,457	35,173	35,173	24,186	36,460	11,408	47,730	18,410	287

Toluene & Xylene mixed 1:3

TX13 2ppm	CH1	CH2	CH3	CH4	CH5	CH6	CH7	CH8	CH9
CH1	5,194	16,955	15,606	12,737	15,492	20,121	13,457	19,189	19,921
CH2	16,955	325	4,041	6,741	2,759	2,575	5,509	1,352	2,769
CH3	15,606	4,041	1,801	5,172	1,468	5,563	4,070	4,591	5,410
CH4	12,737	6,741	5,172	1,790	4,405	8,894	2,473	7,999	8,667
CH5	15,492	2,759	1,468	4,405	941	4,707	3,635	4,008	4,851
CH6	20,121	2,575	5,563	8,894	4,707	367	8,084	1,379	515
CH7	13,457	5,509	4,070	2,473	3,635	8,084	1,670	6,238	7,573
CH8	19,189	1,352	4,591	7,999	4,008	1,379	6,238	307	1,620
CH9	19,921	2,769	5,410	8,667	4,851	515	7,573	1,620	116

TX13 4ppm	CH1	CH2	CH3	CH4	CH5	CH6	CH7	CH8	CH9
CH1	6,006	43,388	37,474	35,309	38,404	48,083	35,470	45,632	47,809
CH2	43,388	687	7,700	10,884	5,371	5,455	9,420	2,558	5,893
CH3	37,474	7,700	2,104	3,938	2,998	12,294	5,482	9,921	12,007
CH4	35,309	10,884	3,938	2,109	5,555	15,352	3,987	13,105	14,866
CH5	38,404	5,371	2,998	5,555	1,026	10,000	5,067	7,583	10,074
CH6	48,083	5,455	12,294	15,352	10,000	325	13,724	3,088	813
CH7	35,470	9,420	5,482	3,987	5,067	13,724	1,853	11,501	13,789
CH8	45,632	2,558	9,921	13,105	7,583	3,088	11,501	529	3,518
CH9	47,809	5,893	12,007	14,866	10,074	813	13,789	3,518	167

TX13 6ppm	CH1	CH2	CH3	CH4	CH5	CH6	CH7	CH8	CH9
CH1	8,708	65,512	56,495	56,912	60,065	73,892	55,333	70,039	73,575
CH2	65,512	845	12,622	12,713	6,188	9,061	12,465	4,916	9,641
CH3	56,495	12,622	2,785	4,113	7,063	20,675	6,643	12,465	20,032
CH4	56,912	12,713	4,113	2,712	6,702	20,689	6,205	17,087	19,872
CH5	60,065	6,188	7,063	6,702	1,374	14,291	6,953	10,556	14,344
CH6	73,892	9,061	20,675	20,689	14,291	337	19,932	4,382	1,230
CH7	55,333	12,465	6,643	6,205	6,953	19,932	2,020	11,501	13,789
CH8	70,039	4,916	12,465	17,087	10,556	4,382	11,501	791	4,999
CH9	73,575	9,641	20,032	19,872	14,344	1,230	13,789	4,999	217

TX13 8ppm	CH1	CH2	CH3	CH4	CH5	CH6	CH7	CH8	CH9
CH1	8,565	88,718	78,516	81,094	83,783	98,729	77,874	94,445	98,563
CH2	88,718	1,015	16,961	14,359	7,339	12,583	14,717	8,022	13,160
CH3	78,516	16,961	3,599	9,826	14,529	28,897	11,893	24,849	28,240
CH4	81,094	14,359	9,826	3,144	11,058	25,644	11,012	21,692	24,887
CH5	83,783	7,339	14,529	11,058	1,317	18,078	10,550	13,568	18,695
CH6	98,729	12,583	28,897	25,644	18,078	340	22,640	5,291	1,637
CH7	77,874	14,717	11,893	11,012	10,550	22,640	2,378	21,718	25,734
CH8	94,445	8,022	24,849	21,692	13,568	5,291	21,718	936	6,593
CH9	98,563	13,160	28,240	24,887	18,695	1,637	25,734	6,593	361

TX13 10ppm	CH1	CH2	CH3	CH4	CH5	CH6	CH7	CH8	CH9
CH1	9,481	105,821	90,973	95,725	100,672	120,679	91,550	9,878	119,959
CH2	105,821	1,243	20,869	14,755	5,873	16,034	16,205	9,876	34,865
CH3	90,973	20,869	3,685	9,329	16,041	35,753	11,509	30,276	34,869
CH4	95,725	14,755	9,329	3,093	9,647	29,584	10,175	24,162	28,515
CH5	100,672	5,873	16,041	9,647	1,205	20,667	10,788	14,789	21,563
CH6	120,679	16,034	35,753	29,584	20,667	349	30,081	6,576	1,506
CH7	91,550	16,205	11,509	10,175	10,788	30,081	2,158	24,726	30,334
CH8	9,878	9,876	30,276	24,162	14,789	6,576	24,726	1,122	7,529
CH9	119,959	34,865	34,869	28,515	21,563	1,506	30,334	7,529	389

Toluene & Xylene mixed 3:1

TX31 2ppm	CH1	CH2	CH3	CH4	CH5	CH6	CH7	CH8	CH9
CH1	1,578	7,758	5,916	5,126	4,724	9,359	6,380	8,592	9,666
CH2	7,758	327	3,482	6,186	4,905	1,779	3,174	1,221	2,199
CH3	5,916	3,482	468	3,988	2,654	4,396	2,339	3,797	4,865
CH4	5,126	6,186	3,988	2,131	3,919	7,589	5,039	6,935	8,062
CH5	4,724	4,905	2,654	3,919	868	5,504	2,768	4,816	5,857
CH6	9,359	1,779	4,396	7,589	5,504	145	4,598	1,104	503
CH7	6,380	3,174	2,339	5,039	2,768	4,598	966	3,019	4,112
CH8	8,592	1,221	3,797	6,935	4,816	1,104	3,019	424	1,393
CH9	9,666	2,199	4,865	8,062	5,857	503	4,112	1,393	165

TX31 4ppm	CH1	CH2	CH3	CH4	CH5	CH6	CH7	CH8	CH9
CH1	3,503	22,518	16,851	16,512	15,575	25,294	19,336	23,887	25,626
CH2	22,518	760	6,633	9,821	8,623	3,926	5,683	2,427	4,670
CH3	16,851	6,633	802	3,777	2,773	9,771	5,031	8,207	10,521
CH4	16,512	9,821	3,777	3,025	4,644	13,030	7,665	11,430	13,775
CH5	15,575	8,623	2,773	4,644	1,237	10,919	5,633	9,566	11,375
CH6	25,294	3,926	9,771	13,030	10,919	171	7,923	1,756	874
CH7	19,336	5,683	5,031	7,665	5,633	7,923	1,552	6,664	7,700
CH8	23,887	2,427	8,207	11,430	9,566	1,756	6,664	609	2,446
CH9	25,626	4,670	10,521	13,775	11,375	874	7,700	2,446	294

TX31 6ppm	CH1	CH2	CH3	CH4	CH5	CH6	CH7	CH8	CH9
CH1	5,081	39,001	30,388	28,896	29,438	43,063	34,135	41,257	43,724
CH2	39,001	1,101	10,021	15,015	11,198	5,931	8,477	3,734	7,092
CH3	30,388	10,021	1,186	5,373	2,706	14,514	7,302	8,477	15,683
CH4	28,896	15,015	5,373	2,096	6,101	19,578	11,341	17,471	20,725
CH5	29,438	11,198	2,706	6,101	1,550	14,740	6,895	13,064	15,750
CH6	43,063	5,931	14,514	19,578	14,740	209	11,605	2,518	1,327
CH7	34,135	8,477	7,302	11,341	6,895	11,605	1,781	6,664	7,700
CH8	41,257	3,734	8,477	17,471	13,064	2,518	6,664	1,045	3,668
CH9	43,724	7,092	15,683	20,725	15,750	1,327	7,700	3,668	446

TX31 8ppm	CH1	CH2	CH3	CH4	CH5	CH6	CH7	CH8	CH9
CH1	7,533	59,317	49,375	48,695	48,924	64,125	53,873	62,045	65,015
CH2	59,317	1,351	13,277	17,410	13,072	8,769	10,235	6,082	10,123
CH3	49,375	13,277	2,088	8,063	6,085	19,988	11,111	17,513	21,267
CH4	48,695	17,410	8,063	2,685	9,749	24,983	15,130	22,473	26,258
CH5	48,924	13,072	6,085	9,749	1,620	19,430	9,963	17,266	20,709
CH6	64,125	8,769	19,988	24,983	19,430	403	13,825	4,099	2,681
CH7	53,873	10,235	11,111	15,130	9,963	13,825	3,001	14,180	15,542
CH8	62,045	6,082	17,513	22,473	17,266	4,099	14,180	1,417	5,706
CH9	65,015	10,123	21,267	26,258	20,709	2,681	15,542	5,706	640

TX31 10ppm	CH1	CH2	CH3	CH4	CH5	CH6	CH7	CH8	CH9
CH1	9,417	78,736	64,186	65,433	65,663	85,851	72,312	6,930	87,644
CH2	78,736	1,710	16,959	19,913	14,387	10,064	10,548	6,930	26,568
CH3	64,186	16,959	2,886	6,516	5,555	24,614	12,111	21,742	26,568
CH4	65,433	19,913	6,516	3,057	8,172	27,890	14,195	24,835	29,871
CH5	65,663	14,387	5,555	8,172	1,379	21,580	9,117	19,172	23,576
CH6	85,851	10,064	24,614	27,890	21,580	573	16,552	3,920	2,332
CH7	72,312	10,548	12,111	14,195	9,117	16,552	2,549	15,842	17,228
CH8	6,930	6,930	21,742	24,835	19,172	3,920	15,842	1,440	6,095
CH9	87,644	26,568	26,568	29,871	23,576	2,332	17,228	6,095	912

Acetone & Toluene & Xylene mixed 1:1:1

ATX111 2ppm	CH1	CH2	CH3	CH4	CH5	CH6	CH7	CH8	CH9
CH1	2,975	12,797	17,225	12,084	10,372	20,790	15,276	16,237	21,917
CH2	12,797	2,568	8,524	5,787	4,914	8,845	3,501	4,763	9,954
CH3	17,225	8,524	1,780	8,226	9,320	13,236	7,743	6,343	14,191
CH4	12,084	5,787	8,226	4,720	6,029	13,239	7,508	7,659	14,396
CH5	10,372	4,914	9,320	6,029	1,673	11,726	5,460	7,271	13,868
CH6	20,790	8,845	13,236	13,239	11,726	680	8,581	7,339	1,634
CH7	15,276	3,501	7,743	7,508	5,460	8,581	2,191	3,723	9,524
CH8	16,237	4,763	6,343	7,659	7,271	7,339	3,723	1,492	8,376
CH9	21,917	9,954	14,191	14,396	13,868	1,634	9,524	8,376	656

ATX111 4ppm	CH1	CH2	CH3	CH4	CH5	CH6	CH7	CH8	CH9
CH1	6,035	35,301	42,345	35,436	33,623	50,950	40,821	43,792	53,287
CH2	35,301	3,564	11,224	6,428	3,805	17,540	7,436	10,819	19,780
CH3	42,345	11,224	2,339	11,379	12,928	24,089	13,472	13,697	26,185
CH4	35,436	6,428	11,379	4,532	6,119	19,871	9,925	11,589	22,236
CH5	33,623	3,805	12,928	6,119	1,825	19,579	7,954	12,189	22,306
CH6	50,950	17,540	24,089	19,871	19,579	1,098	13,185	10,624	3,014
CH7	40,821	7,436	13,472	9,925	7,954	13,185	1,745	5,610	15,921
CH8	43,792	10,819	13,697	11,589	12,189	10,624	5,610	1,757	13,354
CH9	53,287	19,780	26,185	22,236	22,306	3,014	15,921	13,354	745

ATX111 6ppm	CH1	CH2	CH3	CH4	CH5	CH6	CH7	CH8	CH9
CH1	6,791	60,006	69,614	62,574	60,450	84,282	69,511	74,206	87,918
CH2	60,006	5,165	15,196	9,166	4,860	26,642	11,769	16,862	30,201
CH3	69,614	15,196	2,902	16,433	17,271	35,019	20,275	11,769	38,212
CH4	62,574	9,166	16,433	4,918	7,340	26,291	12,242	15,044	29,852
CH5	60,450	4,860	17,271	7,340	1,574	26,134	9,649	15,779	30,379
CH6	84,282	26,642	35,019	26,291	26,134	1,532	17,990	14,511	4,587
CH7	69,511	11,769	20,275	12,242	9,649	17,990	1,730	5,610	15,921
CH8	74,206	16,862	11,769	15,044	15,779	14,511	5,610	2,007	18,833
CH9	87,918	30,201	38,212	29,852	30,379	4,587	15,921	18,833	976

ATX111 8ppm	CH1	CH2	CH3	CH4	CH5	CH6	CH7	CH8	CH9
CH1	11,826	90,850	101,995	96,886	95,469	120,231	103,466	108,690	124,434
CH2	90,850	6,843	21,466	16,720	13,300	36,998	20,310	25,447	41,188
CH3	101,995	21,466	5,010	27,087	27,341	47,393	30,730	31,982	51,081
CH4	96,886	16,720	27,087	5,115	11,767	31,861	16,008	19,146	35,889
CH5	95,469	13,300	27,341	11,767	2,506	31,594	13,702	20,460	36,635
CH6	120,231	36,998	47,393	31,861	31,594	1,878	20,961	17,216	6,759
CH7	103,466	20,310	30,730	16,008	13,702	20,961	2,834	11,663	28,230
CH8	108,690	25,447	31,982	19,146	20,460	17,216	11,663	2,505	24,702
CH9	124,434	41,188	51,081	35,889	36,635	6,759	28,230	24,702	1,211

ATX111 10ppm	CH1	CH2	CH3	CH4	CH5	CH6	CH7	CH8	CH9
CH1	14,255	113,053	128,909	125,730	124,224	156,628	133,813	30,601	162,967
CH2	113,053	6,700	22,219	18,965	15,180	46,697	24,034	30,600	63,387
CH3	128,909	22,219	6,383	30,327	30,209	57,733	35,577	37,531	63,387
CH4	125,730	18,965	30,327	5,443	8,347	34,845	13,871	18,660	40,763
CH5	124,224	15,180	30,209	8,347	2,578	34,568	10,537	19,268	41,841
CH6	156,628	46,697	57,733	34,845	34,568	2,277	26,031	21,195	7,921
CH7	133,813	24,034	35,577	13,871	10,537	26,031	1,720	10,255	33,255
CH8	30,601	30,600	37,531	18,660	19,268	21,195	10,255	2,818	28,915
CH9	162,967	63,387	63,387	40,763	41,841	7,921	33,255	28,915	1,273

Supplementary Table 7. Euclidean distance for similarity analysis between all measurement data of intra-class for the exhaust gases and their individual components.

NO	CH1	CH2	CH3	CH4	CH5	CH6	CH7	CH8	CH9
CH1	9,226	184,444	180,122	113,617	152,902	104,064	145,644	52,714	57,173
CH2	184,444	2,553	8,611	72,066	32,553	81,513	39,378	145,208	241,154
CH3	180,122	8,611	4,354	67,359	27,948	77,021	34,851	140,880	236,851
CH4	113,617	72,066	67,359	6,867	41,457	14,531	34,056	82,639	170,147
CH5	152,902	32,553	27,948	41,457	4,990	50,373	14,195	113,897	209,452
CH6	104,064	81,513	77,021	14,531	50,373	7,054	43,437	71,066	160,191
CH7	145,644	39,378	34,851	34,056	14,195	43,437	3,961	110,460	202,281
CH8	52,714	145,208	140,880	82,639	113,897	71,066	110,460	6,538	102,768
CH9	57,173	241,154	236,851	170,147	209,452	160,191	202,281	102,768	12,208

NO_2	CH1	CH2	CH3	CH4	CH5	CH6	CH7	CH8	CH9
CH1	709,152	16,600,531	20,158,253	17,152,995	16,020,773	12,901,260	15,793,178	9,606,147	10,155,534
CH2	16,600,531	169,106	3,557,752	653,872	762,849	3,708,264	834,445	7,025,231	6,466,291
CH3	20,158,253	3,557,752	64,447	3,005,524	4,140,204	7,263,827	4,366,048	10,582,922	10,023,941
CH4	17,152,995	653,872	3,005,524	189,132	1,223,447	4,258,774	1,426,126	7,577,767	7,019,176
CH5	16,020,773	762,849	4,140,204	1,223,447	243,883	3,126,900	714,695	6,445,677	5,887,538
CH6	12,901,260	3,708,264	7,263,827	4,258,774	3,126,900	465,892	2,971,793	3,328,363	2,950,715
CH7	15,793,178	834,445	4,366,048	1,426,126	714,695	2,971,793	244,476	6,218,002	5,837,987
CH8	9,606,147	7,025,231	10,582,922	7,577,767	6,445,677	3,328,363	6,218,002	509,387	902,735
CH9	10,155,534	6,466,291	10,023,941	7,019,176	5,887,538	2,950,715	5,837,987	902,735	404,143

CO	CH1	CH2	CH3	CH4	CH5	CH6	CH7	CH8	CH9
CH1	5,716	6,850	10,953	9,667	7,870	10,256	8,255	7,425	7,137
CH2	6,850	4,908	10,785	8,746	7,499	9,511	8,036	7,318	7,409
CH3	10,953	10,785	6,531	10,723	12,039	11,812	8,152	15,686	15,645
CH4	9,667	8,746	10,723	8,697	8,613	8,780	8,368	11,602	12,210
CH5	7,870	7,499	12,039	8,613	6,523	8,835	8,812	7,965	8,921
CH6	10,256	9,511	11,812	8,780	8,835	9,283	9,245	11,862	12,655
CH7	8,255	8,036	8,152	8,368	8,812	9,245	6,998	11,795	11,817
CH8	7,425	7,318	15,686	11,602	7,965	11,862	11,795	4,412	4,690
CH9	7,137	7,409	15,645	12,210	8,921	12,655	11,817	4,690	4,310

CO_2	CH1	CH2	CH3	CH4	CH5	CH6	CH7	CH8	CH9
CH1	7,025	9,193	10,234	10,961	11,028	8,188	9,518	8,292	7,847
CH2	9,193	2,705	2,579	3,456	3,810	4,737	3,016	4,457	3,889
CH3	10,234	2,579	2,022	2,755	3,333	5,228	2,654	5,148	4,474
CH4	10,961	3,456	2,755	2,883	3,434	6,347	3,277	5,591	5,205
CH5	11,028	3,810	3,333	3,434	3,749	6,523	3,873	5,621	5,443
CH6	8,188	4,737	5,228	6,347	6,523	687	5,663	4,729	5,348
CH7	9,518	3,016	2,654	3,277	3,873	5,663	3,187	5,069	4,337
CH8	8,292	4,457	5,148	5,591	5,621	4,729	5,069	4,523	4,667
CH9	7,847	3,889	4,474	5,205	5,443	5,348	4,337	4,667	4,382

Diesel	CH1	CH2	CH3	CH4	CH5	CH6	CH7	CH8	CH9
CH1	48,302	526,662	448,497	251,766	110,511	199,476	352,028	369,712	374,140
CH2	526,662	5,953	78,181	276,954	580,597	345,156	175,251	161,913	170,269
CH3	448,497	78,181	11,118	47,737	147,468	93,218	56,066	122,793	218,725
CH4	251,766	276,954	198,959	30,765	104,432	58,813	42,950	107,021	175,718
CH5	110,511	580,597	502,577	312,086	55,262	54,445	145,451	32,352	73,631
CH6	199,476	345,156	266,983	74,649	273,834	43,955	97,665	50,988	125,711
CH7	352,028	175,251	97,434	106,299	408,104	170,682	20,345	143,071	216,502
CH8	369,712	161,913	125,038	175,918	422,282	207,516	118,770	23,111	98,139
CH9	374,140	170,269	145,734	183,352	425,878	212,403	129,335	41,263	19,199

Gasoline	CH1	CH2	CH3	CH4	CH5	CH6	CH7	CH8	CH9
CH1	10,323	121,295	161,043	143,335	46,144	87,435	179,978	40,047	61,776
CH2	121,295	14,280	135,115	88,886	78,586	84,757	104,169	87,701	141,643
CH3	161,043	135,115	13,851	47,737	147,468	93,218	56,066	122,793	218,725
CH4	143,335	88,886	47,737	12,228	104,432	58,813	42,950	107,021	175,718
CH5	46,144	78,586	147,468	104,432	10,340	54,445	145,451	32,352	73,631
CH6	87,435	84,757	93,218	58,813	54,445	10,628	97,665	50,988	125,711
CH7	179,978	104,169	56,066	42,950	145,451	97,665	15,721	143,071	216,502
CH8	40,047	87,701	122,793	107,021	32,352	50,988	143,071	7,736	98,139
CH9	61,776	141,643	218,725	175,718	73,631	125,711	216,502	98,139	7,688

$CO+CO_2$	CH1	CH2	CH3	CH4	CH5	CH6	CH7	CH8	CH9
CH1	5,942	9,900	12,706	9,372	5,817	7,348	6,835	6,629	7,685
CH2	9,900	3,082	4,557	3,283	8,347	4,999	5,470	6,437	13,748
CH3	12,706	4,557	2,106	5,310	11,157	7,612	7,702	9,188	16,631
CH4	9,372	3,283	5,310	3,556	7,879	4,819	5,263	6,049	13,153
CH5	5,817	8,347	11,157	7,879	5,362	5,943	5,827	5,483	8,347
CH6	7,348	4,999	7,612	4,819	5,943	4,427	4,655	4,840	10,692
CH7	6,835	5,470	7,702	5,263	5,827	4,655	4,183	5,209	10,231
CH8	6,629	6,437	9,188	6,049	5,483	4,840	5,209	4,819	9,562
CH9	7,685	13,748	16,631	13,153	8,347	10,692	10,231	9,562	7,420

$CO+CO_2+NO$	CH1	CH2	CH3	CH4	CH5	CH6	CH7	CH8	CH9
CH1	5,942	9,900	12,706	9,372	5,817	7,348	6,835	6,629	7,685
CH2	9,900	3,082	4,557	3,283	8,347	4,999	5,470	6,437	13,748
CH3	12,706	4,557	2,106	5,310	11,157	7,612	7,702	9,188	16,631
CH4	9,372	3,283	5,310	3,556	7,879	4,819	5,263	6,049	13,153
CH5	5,817	8,347	11,157	7,879	5,362	5,943	5,827	5,483	8,347
CH6	7,348	4,999	7,612	4,819	5,943	4,427	4,655	4,840	10,692
CH7	6,835	5,470	7,702	5,263	5,827	4,655	4,183	5,209	10,231
CH8	6,629	6,437	9,188	6,049	5,483	4,840	5,209	4,819	9,562
CH9	7,685	13,748	16,631	13,153	8,347	10,692	10,231	9,562	7,420
